# The Rapid Screening of Triazophos Residues in Agricultural Products by Chemiluminescent Enzyme Immunoassay

**DOI:** 10.1371/journal.pone.0133839

**Published:** 2015-07-28

**Authors:** Ge Chen, Lihua Yang, Maojun Jin, Pengfei Du, Chan Zhang, Jian Wang, Hua Shao, Fen Jin, Lufei Zheng, Shanshan Wang, Yongxin She, Jing Wang

**Affiliations:** Key Laboratory for Agro-Products Quality and Safety, Institute of Quality Standards & Testing Technology for Agro-Products, Chinese Academy of Agricultural Sciences, Beijing, 100081, China; Ghent University, BELGIUM

## Abstract

A highly sensitive chemiluminescent enzyme immunoassay (CLEIA) method was developed in this study for efficient screening of triazophos residues in a large number of samples. Based on the maximum residue limits (MRLs) set by China and CAC for triazophos in different agro-products, the representative apple, orange, cabbage, zucchini, and rice samples were selected as spiked samples, and the triazophos at the concentrations of the MRL values were spiked to blank samples. Subsequently, the five samples with the spiked triazophos standard were measured by CLEIA 100 times, and the detection results indicated that the correction factors of the apple, orange, cabbage, zucchini, and rice were determined as 0.79, 0.66, 0.85, 0.76, and 0.91, respectively. In this experiment, 1500 real samples were detected by both the CLEIA and the GC-MS methods. With the GC-MS method, 1462 samples were identified as negative samples and 38 samples as positive samples. Based on the correction factors, the false positive rate of the CLEIA method was 0.13%, and false negative rate was 0. The results showed that the established CLEIA method could be used to screen a large number of real samples.

## Introduction

In recent years, commercial test kits and test strips for rapid detection of pesticide residues[1–2] with the easy, inexpensive and visually discernible characteristics can be directly applied to the entire procedure of food production, processing and sales, which meet the requirements of on-site quick pesticide residue[3] monitoring management to some extent. The Environmental Protection Agency (EPA), American Association of Official Analytical Chemists (AOAC), and Ministry of Agriculture Food Safety and Inspection Division (FSIS) have presented guidelines for the evaluation and approval of immunoassay kits for pesticide residue in agricultural products. The test kits and strips for commercial use are widely used in many countries [4–5]. Currently, enzyme-linked immunosorbent assay (ELISA) has been widely applied to pesticide residue testing [6–8]. Although the ELISA method has high sensitivity in standard detection, some of the lowest detection limits of pesticides in the samples are still less than their respective maximum residue level (MRL) values. Chemiluminescent enzyme immunoassay (CLEIA) [9], which has technical advantages and has been successfully applied in the medical field, is expected to apply to the field of pesticide residue detection [10–13]. However, CLEIA in pesticide residue screening is not yet mature, and we need to further our study to verify its effect and promote the applications of the CLEIA detection methods in this field.

The CLEIA method has different responses to various matrices. When this method was applied to different matrices of the same quality, luminescence signals may be different. To make the luminescence signals accurately indicate the contents of the analytes, the luminescence signals of the analytes of known quantities must be first measured by the CLEIA method to calculate the quantitative correction factor. The instrumental analytical methods have the following disadvantages: Complicated concentration and derivatization steps are needed to obtain desired sensitivity; heavy use of toxic solvents is harmful to operators; skilled analysts and expensive instruments are required. As the precision of the CLEIA method is not as good as the instrumental analytical methods, the CLEIA method for pesticide residue detection can be used as a method for screening a large number of samples [14]. A great merit of CLEIA is to justify whether a sample is negative or positive so that a large proportion of samples could prevent detection by the instrumental analytical methods.

The study on how to ensure the screening precision of the CLEIA method was carried out in our previous study [15–16]. To analyze and evaluate the detection quality of CLEIA, the false negative rate of triazophos was investigated. This provides reference for pesticide residue detection personnel and agricultural product manufacturing enterprises or individuals. This study determined the correction factors of the CLEIA method for different matrices by selecting the agricultural products (apple, orange, cabbage, zucchini, and rice) that have been registered for triazophos pesticides. According to the MRLs set by China and CAC for pesticide residues, the spiked concentrations were determined for different agricultural products. The selected agricultural products were used as blank samples with triazophos at restricted concentrations added, and then the CLEIA method was used to test the triazophos concentrations in the spiked samples. Each test was repeated 100 times to obtain the correction factor (f) of each sample. Later, the triazophos concentrations in real samples were measured by GC-MS to determine the negative and positive samples. After the results obtained by the two methods were compared, the false negatives and false positives were obtained for the corrected CLEIA method. The false negatives and false positives determined by the CLEIA method were low, and therefore, CLEIA is suitable for screening triazophos pesticides in real agricultural products.

## Materials and Methods

### Immunoreagents, Materials and Instruments

Blank matrices of the apple, orange, cabbage, zucchini and rice were purchased from local supermarkets, which are of high quality and pollution-free, as confirmed by GC-MS.

The randomly selected 300 real samples of the apple, orange, cabbage, zucchini, and rice were purchased from Carrefour, Wal-Mart, other supermarkets, and farmers markets, totaling 1500 samples.

The following instruments were prepared: Multifunctional analytic detector (Promega, WI, USA); high-speed refrigerated centrifuge (Thermo Fisher, MA, USA); 1–1000 μL pipette (Eppendorf, Germany); white opaque 96 flat-bottomed well plate (COSTAR, NY, USA); Agilent 7890A gas chromatography-tandem mass spectrometry (Agilent, CA, USA).

The triazophos standard, Luminol, 4 - (-1- base)—imidazole phenol (4-IMP), N,N-dimethylformamide(DMF), N-hydroxysuccinimide(NHS), N,N'-dicyclohexyl carbodiimide(DCC) and horseradish peroxidase(HRP) were purchased from Sigma-Aldrich (St. Louis MO, USA). PSA and C_18_ solid phase extraction packing materials were purchased from Bonna-Agela Technologies (Tianjin, China). Acetonitrile and methanol of the HPLC grade were got from Thermo Fisher Scientific (MA, USA). All other chemicals and organic solvents, including Tris, hydrochloric acid, Tween 20, peroxide, were of analytical grade or better and were purchased from Beijing chemical industry group Co. Ltd (Beijing, China).

**Fig 1:**
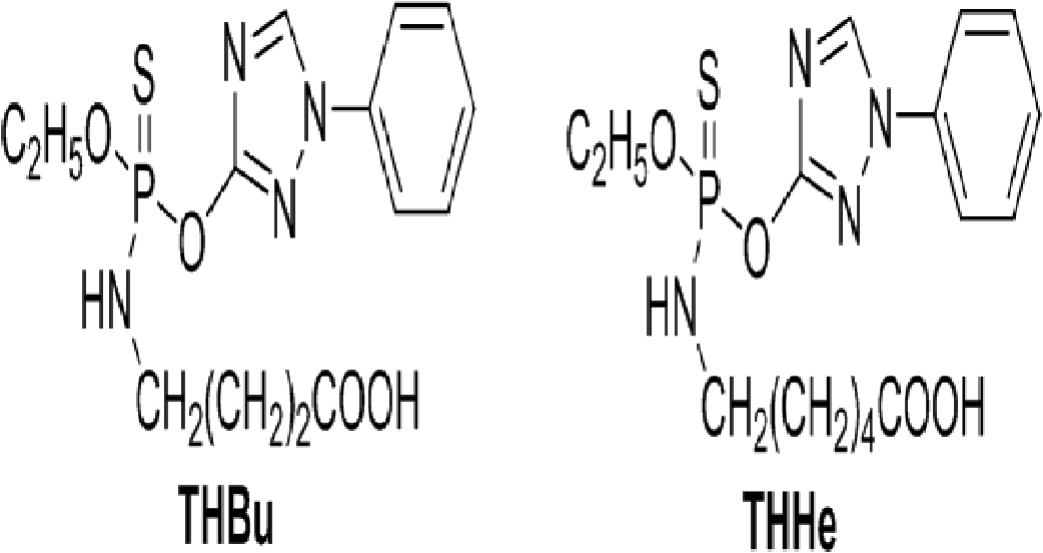
The molecular structures of two haptens for triazophos.

The synthesis of haptens[17–19] (The molecular structures of THHe and THHu were given in Fig 1) and the preparation of monoclonal antibody have been described in the previous paper. Briefly, BALB/c female mice (8–10 weeks of age) were immunized subcutaneously with BSA–THHe conjugate (100 μg) in phosphate-buffered saline (PBS) and complete Fround’s adjuvant 1:1 (v/v) initially and subsequently using incomplete Fround’s adjuvant at 2 week intervals three times. One week after the last injection, mice were tail bled to check for antibody activity. Once this was found to be positive, the mouse was given one more booster injection with 100 μg of conjugate in PBS (without adjuvant), it was killed 3 days later, its spleen was removed, and the spleen cells were fused with SP2/0 murine myeloma cells. Cell fusion procedures were carried out essentially. Culture supernatants were screened for the presence of antibodies that recognized triazophos 12–14 days after cell fusion. The screening consisted of the simultaneous performance of noncompetitive and competitive indirect ELISAs to test the ability of antibodies to bind the hapten–OVA conjugate and to recognize triazophos. Selected hybridomas were cloned by limiting dilution using HT media on a feeder layer of BALB/c thymocytes (~10^6^ cells/well) and peritoneal macrophages (~5000 cells/well). Stable antibody-producing clones were expanded and cryopreserved in liquid nitrogen. After titer testing with indirect ELISA of the mice ascites, the McAbs were separated by the method of salting out (with caprylic acid-ammonium sulfate) and then were purified by an Immuno-Pure (A) IgG purification kit (Pierce, USA) [20–21].

### Preparation of the Buffer Solution

Coating buffer: 0.05 mol/L pH 7.2 Tris-HCl buffer diluted antibodies; washing buffer: 0.01 mol/L pH 7.4 PBS buffer containing 0.05% Tween 20 (PBST); competitive reaction buffer: 0.05 mol/L pH 7.2 Tris-HCl buffer; sensitizing buffer: 0.05 mol/L pH 9.0 Tris-HCl buffer.

Triazophos standard solution: 0.0l g accurately weighed triazophos standard (±0.00001 g) was dissolved in methanol and the volume was set to 100 mL to obtain 100 mg/L standard stock solution, which was then stored in the dark at 4°C. The competitive reaction buffer was used to dilute the triazophos standard stock solution into standard working solutions of different concentrations. The procedure was repeated 10 times, and then the average inhibition rates were measured for each concentration and a CLEIA standard curve was established. Subsequently, the working solution was prepared extemporaneously.

### Detection Procedures of Direct Competitive CLEIA

In this study, we used direct competitive CLEIA with a high sensitivity and stability [22–24]. The 0.05 mol/L pH 7.2 Tris-HCl buffer was used to dilute the triazophos antibody to the concentration of 2μg/mL (100 μL/well) and added to the COSTAR white opaque plate, which was placed into a constant temperature humidity chamber (temperature 37°C; humidity 75%) and coated for 2 h. The coated opaque plate was moved out first, and then the temperature returned to the ambient room temperature. After the plate was washed three times with PBST (PBS containing 0.05% Tween 20, pH 7.4), free binding sites of the wells were blocked with 300 μL/well of 1% Tryptone in 50 mM Tris-HCl buffer (pH 7.2) for 30 min at 37°C. Then, the standard (or sample, 50 μL/well) diluted with the competitive reaction buffer and the enzyme-hapten at the concentration of 0.006 μg/mL (50 μL/well) were added in sequence and incubated for 1 h. After the white opaque plate was removed, the solution was washed at room temperature, and 200 μL/well luminescent sensitizing solution was added and kept in dark for 5 min. The chemiluminometric signal generated from the HRP-luminol-H_2_O_2_ system was measured with the Promega chemiluminescent detector. The luminous intensity is represented as relative light units (RLU).

### Sample Preparation

Blank samples (apple, orange, cabbage, zucchini, and rice) were mashed and then stored in a refrigerator at -20°C. Ten grams of rice blank sample (accurate to 0.01 g) and 3 mL of water were added into a 50 mL plastic centrifuge tube. The acetonitrile extraction solution was added to the blank sample to obtain a constant volume of 10 mL, and then it was homogenized for 0.5 min at a high speed; subsequently, 4.0 g anhydrous MgSO_4_ and 1.0 g NaCl were added to it for high-speed vortex for 1 min; finally, it was centrifuged at 10,000 r/min for 5 min at 4°C. The supernatant was poured into a 50 mL plastic centrifuge tube, and the acetonitrile extraction solution of 2 mL was accurately removed from 10 mL plastic centrifuge tube. Subsequently, 50 mg PSA and 50 mg C_18_ dispersive solid-phase extraction purifier were added, and then it was centrifuged at 10,000 r/min for 5 min under 4°C after high-speed vortex for 0.5 min. The samples prepared in the above procedures were detected in the following two methods in the same manner: In the CLEIA method, 1 mL of the supernatant was blown to dryness at 30°C under nitrogen, and the residue was dissolved in 5 mL 6.4% DMF-competitive reaction buffer solution (sample diluted to a concentration in the CLEIA detection linear range), and the dissolved solution was measured by CLEIA. In the GC-MS method[25–26] 4 mL supernatant was blown to dryness at 30°C under nitrogen, and the residue was dissolved in 4 mL of n-hexane, and the solution was filtered through 0.22 μm organic phase membrane for GC-MS analysis.

### Chromatography—Mass Spectrometry Conditions

Gas chromatography-tandem mass spectrometry (GC-MS) was performed in the following conditions: Column: DB-5MS (30 m × 250 μm × 0.25 μm) silica capillary column; oven temperature procedure: 60°C maintained for 4 min, heated by 30°C/min to 180°C, and then heated to 250°C at the speed of 10°C/min and kept for 4 min; carrier gas: helium, purity ≥ 99.999%, constant voltage mode, and 7.136 psi pressure; injection temperature: 220°C; injection volume: 10 μL; injection mode: splitless injection, and the by-pass valve and septum purge valve opened 1.0 min later; electron impact ionization (EI): 70eV; ion source temperature: 200°C; GC-MS interface temperature: 250°C.

### Test Procedure for CLEIA Detection Correction Factors

Ten grams of (accurately to 0.01 g) a blank matrix (apple, orange, cabbage, zucchini, and rice) were placed in 50 mL plastic centrifuge tubes, and each matrix was in 100 parallel. The triazophos standard solution at the MRL level was added to each blank matrix (200 μg/kg added to fruits, 100 μg/kg added to vegetables, and 50 μg/kg added to rice.). The triazophos content in the spiked matrix was detected by CLEIA. The threshold concentration is C_0_ when the false negative rate is 3%, and C_I_ is the real spiked concentration. In this case, we could obtain the correction factor (f) in the formula: Correction factor (f) = C_0_/C_I_.

### False Negative and False Positive of the CLEIA Method

GC-MS and CLEIA were used to detect the triazophos content in the apple, orange, cabbage, zucchini, and rice samples (a total of 1,500 with 300 of each). The triazophos concentration detected by GC-MS in the real samples is considered as C_a_. If C_a_ of a sample is greater than its MRL value, the sample is positive; if C_a_ of a sample is smaller than its MRL value, the sample is negative.

The triazophos concentration detected by CLEIA in the real samples is considered as C_b_. C_b_ corrected by the correction factor (f) is considered as C_c_. If C_c_ of a positive sample is smaller than its MRL value, the CLEIA detection result detected by is false negative; if C_c_ of a negative sample is greater than its MRL value, the CLEIA detection result is false positive.

The formulas are as follows: C_c_ = C_b_/f; False negative rate I (%) = False negative samples/Total positive samples; False positive rate I (%) = False positive samples/Total negative samples.

## Results and Discussions

### Determination of Dynamic Range and Sensitivity

**Fig 2:**
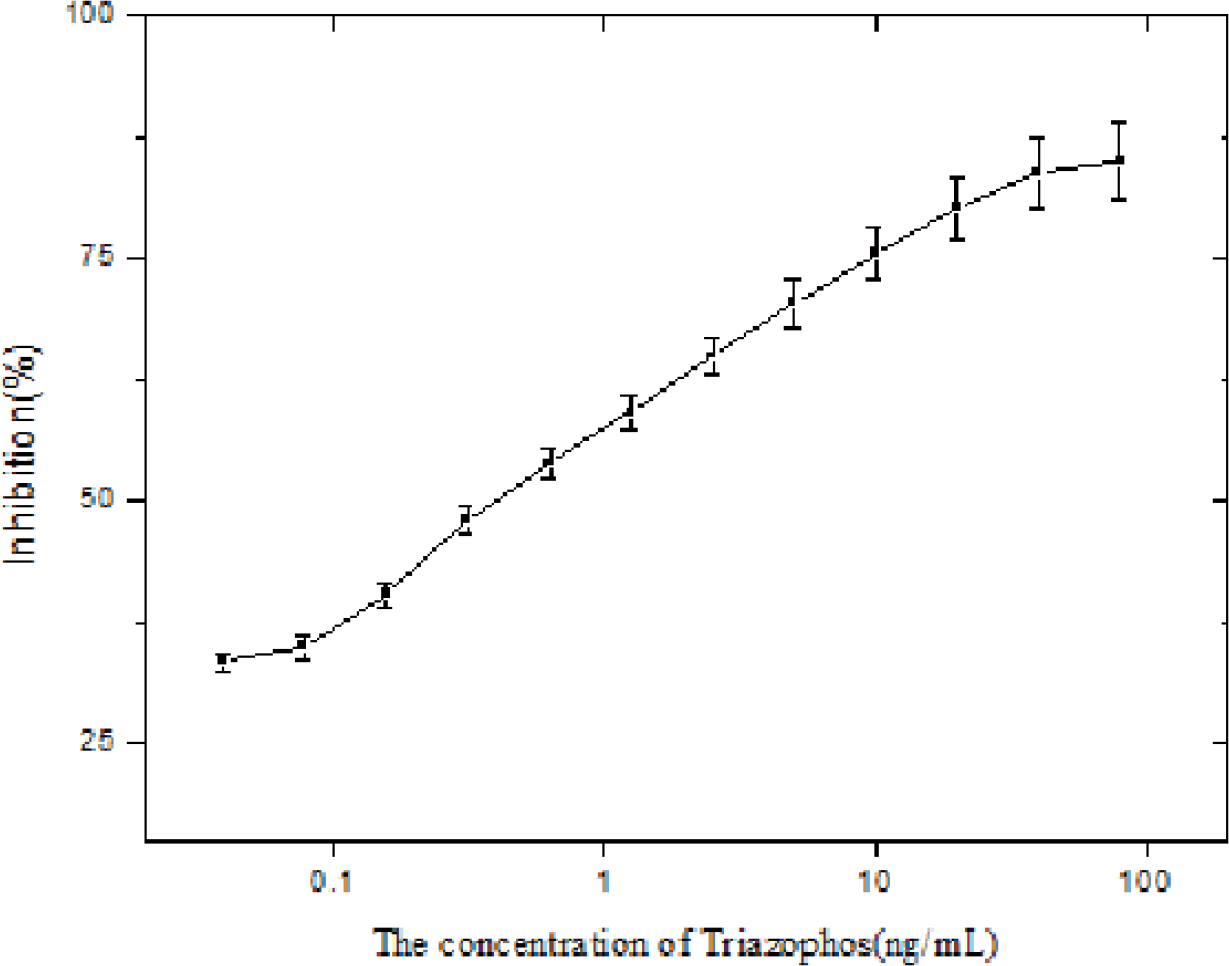
Typical standard curves for triazophos by CLEIA under optimized conditions.

The standard curve for triazophos was established in the concentration range of 0.16–20 ng/mL based on the average of 3 replicate measurements for each concentration. The results were shown in Fig 2. A linear correlation between the inhibition rate and triazophos concentration was obtained between 0.16 and 20 ng/mL. The regression equation was *y* = 8.963Ln(*x*) + 56.40, and the IC_50_ for triazophos was 0.45 ng/mL.

### Correction Factor of the CLEIA Assay for Agricultural Products

Different matrices have different background interference so that the CLEIA sensitivity varies. In order to make the CLEIA detection results of the matrices close to those of real samples, the correction factors of a variety of matrices were calculated in large quantities of experiments. Based on the triazophos MRLs drafted by China and CAC for different agro-products, the spiked concentrations of five agricultural products were obtained (Table 1). The actual range of every kind of matrix with 100 samples was measured by CLEIA to calculate the threshold C_0_ and the correction factor (f) at the false negative rate of 3% (Table 1). The actual ranges of the apple, orange, cabbage, zucchini, and rice matrices measured by CLEIA were 136.24 ~ 249.06 μg/kg, 107.66 ~ 239.40 μg/kg, 79.02 ~ 132.56 μg/kg, 71.36 ~ 140.16 μg/kg, and 17.58 ~ 26.66 μg/kg, respectively, and their thresholds are 157.80 μg/kg, 132.02 μg/kg, 84.68 μg/kg, 76.34 μg/kg, and 18.2 μg/kg, respectively. Based on the experiments, the obtained correction factors of the apple, orange, cabbage, zucchini, and rice were 0.79, 0.66, 0.85, 0.76, and 0.91, respectively. The correction factor of the orange is 0.66, which is the smallest. The result demonstrated that the orange matrix can cause large deviations. This may be due to the complicated orange matrix, causing interference to the chemiluminescence system. In contrast, the correction factor of the rice is largest among the five kinds of matrices, and it has the minimal interference to the chemiluminescence system.

**Table 1:** Correction factors for the CLEIA method.

Sample	MRL (μg/kg)	Spiked concentration C_I_ (μg/kg)	Actual range of detection (μg/kg)	Threshold C_0_ (μg/kg)	f (C_0_/C_I_)
**Apple**	200	200	136.24~249.06	157.80	0.79
**Citrus**	200	200	107.66~239.40	132.02	0.66
**Cabbage**	100	100	79.02~132.56	84.68	0.85
**Zucchini**	100	100	71.36~140.16	76.34	0.76
**Rice**	50	50	17.58~26.66	18.2	0.91

f is the abbreviation of correction factor

### Analysis of False Negative and False Positive by the CLEIA Method

To determine whether CLEIA can be used for rapid screening of large quantities of agricultural products, this study selected the agricultural products (apple, orange, cabbage, zucchini, and rice) that have been registered for triazophos pesticides to analyze the false negative and false positive rates of the CLEIA method. Five types of matrices, for each of which 300 samples were randomly selected from the supermarket, were detected and analyzed by GC-MS and CLEIA, respectively. The detection results of CLEIA need to be corrected by the correction factors. After the test results of GC-MS and results of corrected CLEIA were analyzed, the false negatives and false positives of CLEIA could be obtained.

As shown in Table 2, the negative and positive samples measured by GC-MS and CLEIA are listed in the 3^rd^ and 5^th^ columns, and the false positive and false negative samples are listed in the 4^th^ and 6^th^ columns. For each of the 5 types of samples, the false negative samples in the 6^th^ column could be calculated by comparing the uncorrected CLEIA results with the GC-MS results, and the results are shown in the middle row. The results showed only one false negative sample in 300 apples, two false negative samples in 300 oranges, and two false negative samples in 300 cabbages. In total, 5 false negatives were found in the 1500 samples. However, no false positives were found. By comparing the corrected CLEIA results (bottom row for each type of sample) with the GC-MS results (top row), we could obtain the false positive samples, as listed in the 4^th^ column. The results showed only one false positive in all the 300 apple samples and one in all the 300 zucchini samples. No false negatives were found.

**Table 2:** Agricultural products by CLEIA detection with the false negative and false positive.

Sample	Method	Negative sample	False positive	Positive sample	False negative	Rate of false negative	Rate of false positive
**Apple**	GC-MS	292	——	8	——	——	——
	CLEIA	293	0	7	1	12.50%	0
	Corrected CLEIA	291	1	9	0	0	0.34%
**Citrus**	GC-MS	293	——	7	——	——	——
	CLEIA	295	0	5	2	28.57%	0
	Corrected CLEIA	293	0	7	0	0	0
**Cabbage**	GC-MS	294	——	6	——	——	——
	CLEIA	296	0	4	2	33.33%	0
	Corrected CLEIA	294	0	6	0	0	0
**Zucchini**	GC-MS	291	——	9	——	——	——
	CLEIA	291	0	9	0	0	0
	Corrected CLEIA	290	1	10	0	0	0.34%
**Rice**	GC-MS	292	——	8	——	——	——
	CLEIA	292	0	8	0	0	0
	Corrected CLEIA	292	0	8	0	0	0
**Total of the samples**	GC-MS	1462	——	38	——	——	——
	CLEIA	1467	0	33	5	13.16%	0
	Corrected CLEIA	1460	2	40	0	0	0.13%

In summary, the contents of triazophos in the 1500 agricultural products were measured by GC-MS and CLEIA. The corrected CLEIA detection results showed that only one false positive existed in all the 300 apple samples and one false positive in all the 300 zucchini samples and no false positive samples were identified among the 1500 samples (The determination data were given in S1 Text, S2 Text, S3 Text, S4 Text and S5 Text). This study showed 1462 negative samples and 38 positive samples in 1500 agricultural products by GC-MS. The uncorrected CLEIA results showed 5 false negative samples and no false positive in the 1500 samples, whereas the corrected CLEIA results showed 2 false positive samples and no false negative in the 1500 samples. The uncorrected CLEIA results show that the rates of the false positive and false negative are 0 and 13.16%, respectively. The corrected CLEIA results show that the rates of the false negative and false positive are 0 and 0.13%, respectively, indicating that CLEIA detection has better applications in rapid screening after being corrected by the correction factors.

## Conclusion

The agricultural products registered for triazophos residues (apple, orange, cabbage, zucchini and rice) were selected, and the five samples with the spiked triazophos standard were measured by CLEIA 100 times separately. Based on the detection, the correction factors (f) of the apple, orange, cabbage, zucchini, and rice were acquired as 0.79, 0.66, 0.85, 0.76, and 0.91, respectively.

The triazophos contents of the 1500 agricultural product samples were detected with the GC-MS method and CLEIA in this study. The corrected CLEIA results showed only one false positive in 300 apple samples and one false positive in 300 zucchini samples, and no false negative samples were found in all 5 kinds of samples.

The GC-MS results showed 38 positive samples and 1462 negative samples among the 1500 samples. The corrected CLEIA results showed that the false negative rate is 0 and the false positive rate is 0.13%, demonstrating that corrected CLEIA detection has better applications in rapid screening compared with uncorrected CLEIA

## Supporting Information

S1 Text The determinations of apple real samples by GC-MS and CLEIA. (DOCX)

S2 Text The determinations of orange real samples by GC-MS and CLEIA. (DOCX)

S3 Text The determinations of cabbage real samples by GC-MS and CLEIA. (DOCX)

S4 Text The determinations of zucchinis real samples by GC-MS and CLEIA. (DOCX)

S5 Text The determinations of rice real samples by GC-MS and CLEIA. (DOCX)
