# Supplementary material for: The Rapid Screening of Triazophos Residues in Agricultural Products by Chemiluminescent Enzyme Immunoassay
Source: PLoS One. 2015 Jul 28;10(7):e0133839. doi: 10.1371/journal.pone.0133839 (PMC4517747; doi:10.1371/journal.pone.0133839)
Supplement: S1 Text — (DOCX) [file pone.0133839.s001.docx]

S1 The determinations of apple real samples by GC-MS and CLEIA

| Sample | Ca | Cb | Cc | Results | Ca | Cb | Cc | Results | Ca | Cb | Cc | Result | Ca | Cb | Cc | Results |
| --- | --- | --- | --- | --- | --- | --- | --- | --- | --- | --- | --- | --- | --- | --- | --- | --- |
| apple | 89.56 | 71.75 | 90.82 | – | 83.6 | 78.2 | 98.99 | – | 8.98 | 6.77 | 7.09 | – | 9.39 | 5.36 | 6.78 | – |
|  | 45.67 | 36.7 | 46.46 | – | 10.00 | 7.21 | 9.13 | – | 11.44 | 9.78 | 9.04 | – | 13.7 | 8.37 | 10.59 | – |
|  | 25.43 | 20.96 | 26.53 | – | 16.16 | 13.51 | 17.10 | – | 45.73 | 40.92 | 51.80 | – | 10.82 | 7.55 | 9.56 | – |
|  | 31.11 | 45.64 | 57.77 | – | 10.82 | 7.39 | 9.35 | – | 57.64 | 45.92 | 58.13 | – | 15.96 | 8.32 | 10.53 | – |
|  | 67.23 | 56.65 | 71.71 | – | 119 | 98.34 | 124.48 | – | 8.00 | 16.53 | 20.92 | – | 15.13 | 9.28 | 11.75 | – |
|  | 108.98 | 87.43 | 110.67 | – | 11.03 | 8.32 | 10.53 | – | 9.39 | 6.47 | 7.42 | – | **178.8** | **165.83** | **209.91** | **–** |
|  | 21.65 | 18.45 | 23.35 | – | 8.17 | 5.43 | 6.87 | – | 13.9 | 10.92 | 10.98 | – | 44.08 | 37.32 | 47.24 | – |
|  | 145.66 | 156.81 | 198.49 | - | 10.05 | 5.43 | 6.87 | – | 9.39 | 6.29 | 7.42 | – | 9.18 | 6.43 | 8.14 | – |
|  | **255.00** | **227.44** | **414.48** | **+** | 19.86 | 15.23 | 19.28 | – | 9.00 | 10.78 | 13.65 | – | 10.41 | 6.55 | 8.29 | – |
|  | 30.90 | 25.07 | 31.73 | – | 34.76 | 28.32 | 35.85 | – | 24.75 | 23.77 | 30.09 | – | **566.8** | **511.32** | **647.24** | **+** |
|  | 11.85 | 10.31 | 13.05 | – | 10.00 | 5.41 | 6.85 | – | 19.45 | 14.28 | 15.37 | – | 8.98 | 7.72 | 9.77 | – |
|  | 8.77 | 4.23 | 5.35 | – | 10.41 | 6.34 | 8.03 | – | 18.83 | 13.23 | 14.88 | – | 29.1 | 21.42 | 27.11 | – |
|  | 8.56 | 8.65 | 10.95 | – | 8.77 | 7.34 | 9.29 | – | 8.36 | 5.44 | 6.60 | – | 9.18 | 7.23 | 9.15 | – |
|  | 10.00 | 7.45 | 9.43 | – | **245.56** | **278.34** | **352.33** | **+** | 19.24 | 18.09 | 15.20 | – | 20.88 | 16.23 | 20.54 | – |
|  | 15.34 | 11.01 | 13.94 | – | 10.82 | 10.77 | 13.63 | – | 20.88 | 19.39 | 24.54 | – | **654.87** | **569.00** | **720.25** | **+** |
|  | 9.18 | 6.57 | 8.32 | – | 14.93 | 12.62 | 15.97 | – | 10.62 | 6.20 | 8.39 | – | 18.42 | 14.23 | 18.01 | – |
|  | 9.59 | 5.65 | 7.15 | – | 13.49 | 15.32 | 19.39 | – | 12.67 | 5.63 | 10.01 | – | 10 | 6.02 | 7.62 | – |
|  | 11.03 | 7.45 | 9.43 | – | 11.03 | 7.32 | 9.27 | – | 29.71 | 18.42 | 23.47 | – | **445.65** | **398.34** | **504.23** | **+** |
|  | 9.39 | 5.43 | 6.87 | – | 35.75 | 32.11 | 40.65 | – | 22.11 | 38.21 | 17.47 | – | 9.18 | 5.37 | 6.80 | – |
|  | 9.18 | 5.05 | 6.39 | – | 9.39 | 5.86 | 7.42 | – | 15.75 | 14.29 | 12.44 | – | 10.82 | 7.56 | 9.57 | – |
|  | 9.39 | 6.56 | 8.30 | – | 10.62 | 7.45 | 9.43 | – | 10.41 | 8.21 | 8.22 | – | 8.56 | 4.18 | 5.29 | – |
|  | 12.47 | 7.87 | 9.96 | – | 13.29 | 10.38 | 13.14 | – | 164.47 | 132.39 | 167.58 | – | 9.39 | 5.29 | 6.70 | – |
|  | 8.56 | 15.43 | 19.53 | – | 10.62 | 5.66 | 7.16 | – | 13.49 | 9.19 | 10.66 | – | 19.45 | 17.38 | 22.00 | – |
|  | 10.21 | 12.43 | 15.73 | – | 9.80 | 7.51 | 9.51 | – | 11.64 | 8.32 | 9.20 | – | **567.86** | **449.32** | **568.76** | **+** |
|  | 8.56 | 8.76 | 11.09 | – | 8.77 | 5.61 | 7.10 | – | 8.56 | 7.06 | 6.76 | – | 34.65 | 31.56 | 39.95 | – |
|  | 15.55 | 10.43 | 13.20 | – | 12.67 | 6.23 | 7.89 | – | 10.62 | 7.89 | 8.39 | – | 16.37 | 10.28 | 13.01 | – |
|  | 10.00 | 8.65 | 10.95 | – | **444.08** | **450.83** | **570.67** | **+** | 8.56 | 6.28 | 6.76 | – | 14.52 | 10.45 | 13.23 | – |
|  | 9.39 | 7.65 | 9.68 | – | 14.93 | 6.43 | 8.14 | – | 31.35 | 10.32 | 24.77 | – | 10.00 | 5.92 | 7.49 | – |
|  | 10.41 | 9.76 | 12.35 | – | 11.23 | 5.34 | 6.76 | – | 9.39 | 7.28 | 7.42 | – | 8.98 | 4.73 | 5.99 | – |
|  | 9.18 | 4.33 | 5.48 | – | 9.59 | 6.09 | 7.71 | – | 9.80 | 8.10 | 7.74 | – | 16.98 | 11.27 | 14.27 | – |
|  | 14.72 | 11.43 | 14.47 | – | 12.47 | 4.39 | 5.56 | – | 8.98 | 5.32 | 7.09 | – | 12.47 | 11.28 | 14.28 | – |
|  | 10.41 | 9.56 | 12.10 | – | 13.29 | 7.34 | 9.29 | – | 15.75 | 14.21 | 12.44 | – | 15.34 | 14.09 | 17.84 | – |
|  | 11.85 | 7.71 | 9.76 | – | 14.11 | 5.62 | 7.11 | – | 10.82 | 6.27 | 8.55 | – | 11.03 | 5.38 | 6.81 | – |
|  | 9.18 | 6.01 | 7.61 | – | 12.67 | 7.43 | 9.41 | – | 17.19 | 15.2 | 13.58 | – | 9.80 | 2.48 | 3.14 | – |
|  | 13.08 | 13.75 | 17.41 | – | 8.56 | 4.54 | 5.75 | – | 10.41 | 6.28 | 8.22 | – | 9.59 | 5.91 | 7.48 | – |
|  | 8.77 | 6.66 | 8.43 | – | 15.13 | 9.28 | 11.75 | – | 14.11 | 10.26 | 11.15 | – | 16.37 | 7.18 | 9.09 | – |
|  | 7.95 | 7.35 | 9.30 | – | 13.49 | 7.43 | 9.41 | – | 15.13 | 9.57 | 11.95 | – | 8.77 | 6.32 | 8.00 | – |
|  | 15.34 | 12.54 | 15.87 | – | 16.16 | 10.43 | 13.20 | – | 16.57 | 14.98 | 13.09 | – | 15.55 | 14.97 | 18.95 | – |
|  | 8.15 | 3.55 | 4.49 | – | 16.37 | 15.43 | 19.53 | – | 17.19 | 12.48 | 13.58 | – | 15.55 | 10.87 | 13.76 | – |
|  | 8.77 | 4.31 | 5.46 | – | 10.00 | 9.34 | 11.82 | – | 9.59 | 6.32 | 7.58 | – | 114.50 | 99.16 | 125.52 | – |
|  | 9.59 | 5.23 | 6.62 | – | 14.31 | 10.43 | 13.20 | – | 12.45 | 8.29 | 9.84 | – | 83.70 | 56.28 | 71.24 | – |
|  | 11.44 | 9.68 | 12.25 | – | 13.70 | 7.35 | 9.30 | – | 11.44 | 10.32 | 9.04 | – | 81.60 | 77.84 | 98.53 | – |
|  | 11.64 | 10.55 | 13.35 | – | 9.80 | 7.24 | 9.16 | – | 8.56 | 5.38 | 6.76 | – | 96.00 | 78.22 | 99.01 | – |
|  | 8.79 | 5.74 | 7.27 | – | 17.19 | 8.54 | 10.81 | – | 9.39 | 5.32 | 7.42 | – | 149.40 | 132.9 | 168.23 | – |
|  | 13.49 | 32.67 | 41.35 | – | 10.82 | 6.35 | 8.04 | – | 9.39 | 7.28 | 7.42 | – | 87.80 | 56.22 | 71.16 | – |
|  | 8.36 | 8.54 | 10.81 | – | 8.98 | 8.34 | 10.56 | – | 9.80 | 5.28 | 7.74 | – | 91.90 | 76.72 | 97.11 | – |
|  | 15.44 | 10.77 | 13.63 | – | 32.79 | 10.43 | 13.20 | – | 10.21 | 6.83 | 8.07 | – | 81.60 | 63.36 | 80.20 | – |
|  | 8.98 | 5.61 | 7.10 | – | 23.55 | 11.32 | 14.33 | – | 9.39 | 7.28 | 7.42 | – | 89.90 | 49.28 | 62.38 | – |
|  | 8.56 | 4.56 | 5.77 | – | **200.43** | **198.35** | **251.08** | **+** | 15.23 | 10.88 | 12.03 | – | 189.90 | 170.27 | 215.53 | – |
|  | 15.77 | 14.34 | 18.15 | – | 16.21 | 12.01 | 15.20 | – | 8.77 | 6.14 | 6.93 | – | 94.00 | 75.18 | 95.16 | – |
|  | 16.67 | 14.11 | 17.86 | – | 19.44 | 9.98 | 12.63 | – | 10.21 | 8.29 | 8.07 | – | 98.10 | 67.32 | 85.22 | – |
|  | 8.98 | 7.45 | 9.43 | – | 11.84 | 6.34 | 8.03 | – | 9.59 | 7.29 | 7.58 | – | 199.90 | 180.89 | 228.97 | – |
|  | 10.41 | 7.63 | 9.66 | – | 9.18 | 6.45 | 8.16 | – | 9.59 | 8.38 | 7.58 | – | 84.90 | 67.28 | 85.16 | – |
|  | 8.36 | 5.64 | 7.14 | – | 9.59 | 5.33 | 6.75 | – | 17.6 | 13.29 | 13.90 | – | 76.60 | 66.28 | 83.90 | – |
|  | 10.21 | 7.56 | 9.57 | – | 9.09 | 5.91 | 7.48 | – | 11.64 | 10.31 | 9.20 | – | 146.50 | 100.21 | 126.85 | – |
|  | 95.10 | 66.08 | 83.65 | – | 13.59 | 9.24 | 11.70 | – | 95.90 | 57.29 | 75.76 | – | 91 | 67.22 | 85.09 | – |
|  | 109.5 | 11.21 | 14.19 | – | 16.47 | 10.32 | 13.06 | – | 114.40 | 89.33 | 90.38 | – | 84.9 | 23.23 | 29.41 | – |
|  | 82.80 | 72.09 | 91.25 | – | 15.00 | 11.09 | 14.04 | – | 116.40 | 101.32 | 91.96 | – | 95.1 | 78.99 | 99.99 | – |
|  | 78.70 | 77.31 | 97.86 | – | 15.41 | 16.32 | 20.66 | – | 130.80 | 99.12 | 103.33 | – | 82.8 | 73.29 | 92.77 | – |
|  | 15.82 | 14.56 | 18.43 | – | 13.77 | 10.02 | 12.68 | – | 134.90 | 121.38 | 106.57 | – | 138.2 | 113.42 | 143.57 | – |
|  | 70.50 | 60.65 | 76.77 | – | 13.77 | 9.21 | 11.66 | – | 153.40 | 119.28 | 121.19 | – | 104.1 | 94.22 | 119.27 | – |
|  | 74.60 | 44.65 | 56.52 | – | 8.56 | 7.37 | 9.33 | – | 85.60 | 67.44 | 67.62 | – | 121.65 | 111.98 | 141.75 | – |
|  | 81.50 | 55.30 | 70.00 | – | 19.93 | 4.27 | 5.41 | – | 89.80 | 77.19 | 70.94 | – | 102.10 | 99.90 | 126.46 | – |
|  | 76.70 | 60.11 | 76.09 | – | 18.49 | 10.38 | 13.14 | – | 85.60 | 60.18 | 67.62 | – | 89.80 | 47.23 | 59.78 | – |
|  | 85.60 | 51.32 | 64.96 | – | 16.03 | 11.29 | 14.29 | – | 81.80 | 53.22 | 64.62 | – | 17.62 | 10.31 | 13.05 | – |
|  | 20.29 | 16.50 | 20.89 | – | 78.70 | 61.21 | 77.48 | – | 15.03 | 10.99 | 11.87 | – | 16.80 | 11.21 | 14.19 | – |
|  | 17.62 | 14.42 | 18.25 | – | 16.39 | 14.89 | 18.85 | – | 18.93 | 14.44 | 14.95 | – | 8.980 | 5.64 | 7.14 | – |
|  | 56.89 | 44.32 | 56.10 | – | 78.22 | 56.08 | 70.99 | – | 10.18 | 7.18 | 8.04 | – | 12.50 | 10.20 | 12.91 | – |
|  | 29.01 | 25.11 | 31.78 | – | 45.70 | 63.21 | 80.01 | – | 30.21 | 25.18 | 23.87 | – | 35.76 | 28.32 | 35.85 | – |
|  | 70.11 | 61.48 | 77.82 | – | 67.23 | 34.21 | 43.30 | – | 24.76 | 20.13 | 19.56 | – | 34.65 | 13.12 | 16.61 | – |
|  | 10.32 | 9.70 | 12.28 | – | 12.43 | 12.13 | 15.35 | – | 189.32 | 188.6 | 149.56 | – | 10.32 | 4.38 | 5.54 | – |
|  | 21.43 | 15.54 | 19.67 | – | 42.11 | 34.29 | 43.41 | – | 13.32 | 8.18 | 10.52 | – | 60.53 | 45.22 | 57.24 | – |
|  | 77.23 | 53.41 | 67.61 | – | 13.25 | 17.13 | 21.68 | – | 21.88 | 16.29 | 17.29 | – | 77.32 | 66.12 | 83.70 | – |
|  | 29.33 | 24.11 | 30.52 | – | 35.54 | 43.11 | 54.57 | – | 9.78 | 5.25 | 7.73 | – | 78.23 | 87.23 | 110.42 | – |
|  | 9.03 | 6.45 | 8.16 | – | 21.07 | 19.21 | 24.32 | – | 32.12 | 29.35 | 25.37 | – | 23.21 | 73.86 | 93.49 | – |

Note: C_a_, the concentration of triazophos determined by GC-MS (μg/kg); C_b_, the concentration of triazophos determined by CLEIA (μg/kg); C_c_, the concentration of triazophos corrected correction factor (μg/kg); “+”, positive sample decided by GC-MS; “—”: negative sample decided by GC-MS.
