# Supplementary material for: The Rapid Screening of Triazophos Residues in Agricultural Products by Chemiluminescent Enzyme Immunoassay
Source: PLoS One. 2015 Jul 28;10(7):e0133839. doi: 10.1371/journal.pone.0133839 (PMC4517747; doi:10.1371/journal.pone.0133839)
Supplement: S2 Text — (DOCX) [file pone.0133839.s002.docx]

S2 The determinations of orange real samples by GC-MS and CLEIA

| Sample | Ca | Cb | Cc | Results | Ca | Cb | Cc | Results | Ca | Cb | Cc | Result | Ca | Cb | Cc | Results |
| --- | --- | --- | --- | --- | --- | --- | --- | --- | --- | --- | --- | --- | --- | --- | --- | --- |
| orange | 134.1 | 91.22 | 138.21 | – | 38.84 | 25.84 | 39.15 | – | 23.1 | 15.32 | 23.21 | – | 10.82 | 8.51 | 12.89 | – |
|  | 43.75 | 39.22 | 59.42 | – | 22.34 | 18.77 | 28.44 | – | 38.84 | 18.32 | 27.76 | – | 12.05 | 9.74 | 14.76 | – |
|  | 21.76 | 16.32 | 24.73 | – | 47.74 | 27.09 | 41.05 | – | 78.01 | 65.33 | 98.98 | – | 8.56 | 6.25 | 9.47 | – |
|  | **346.75** | **228.87** | **346.77** | **+** | 33.9 | 35.22 | 53.36 | – | 110.48 | 98.38 | 149.06 | – | 13.29 | 10.98 | 16.64 | – |
|  | 46.55 | 53.75 | 81.44 | – | 75.99 | 59.07 | 89.50 | – | 110.32 | 79.87 | 121.02 | – | 9.80 | 7.49 | 11.35 | – |
|  | 24.60 | 21.50 | 32.58 | – | 32.11 | 29.78 | 45.12 | – | **230.34** | **198.32** | **300.48** | **+** | 8.98 | 6.67 | 10.11 | – |
|  | 28.90 | 23.98 | 36.33 | – | 47.79 | 40.84 | 61.88 | – | 23.44 | 20.1 | 30.45 | – | 9.18 | 6.87 | 10.41 | – |
|  | 89.21 | 65.22 | 98.82 | – | 22.53 | 18.97 | 28.74 | – | 8.77 | 6.46 | 9.79 | – | 9.59 | 7.28 | 11.03 | – |
|  | 28.20 | 20.43 | 30.95 | – | 31.76 | 28.52 | 43.21 | – | 19.24 | 16.93 | 25.65 | – | 17.39 | 15.08 | 22.85 | – |
|  | 80.23 | 74.23 | 112.47 | – | 151.97 | 130.36 | 197.52 | – | 35.98 | 33.67 | 51.02 | – | 20.06 | 17.75 | 26.89 | – |
|  | 13.38 | 9.58 | 14.52 | – | 13.08 | 9.84 | 14.91 | – | 11.03 | 7.47 | 11.32 | – | 9.39 | 7.08 | 10.73 | – |
|  | 9.07 | 7.71 | 11.68 | – | 9.18 | 5.94 | 9.00 | – | 10.21 | 6.65 | 10.08 | – | 11.23 | 8.92 | 13.52 | – |
|  | 10.51 | 5.86 | 8.88 | – | 9.59 | 6.35 | 9.62 | – | 16.98 | 13.42 | 20.33 | – | **325.97** | **289.88** | **439.21** | **+** |
|  | 7.84 | 4.16 | 6.30 | – | 8.36 | 5.12 | 7.76 | – | 13.49 | 9.93 | 15.05 | – | 13.08 | 10.77 | 16.32 | – |
|  | 11.74 | 11.90 | 18.03 | – | 16.16 | 12.92 | 19.58 | – | 39.16 | 35.60 | 53.94 | – | 8.98 | 6.67 | 10.11 | – |
|  | 7.43 | 4.81 | 7.29 | – | 8.98 | 5.74 | 8.70 | – | 9.39 | 5.83 | 8.83 | – | 19.73 | 17.42 | 26.39 | – |
|  | 6.61 | 5.50 | 8.33 | – | 13.49 | 10.25 | 15.53 | – | 24.37 | 20.81 | 31.53 | – | 15.45 | 13.14 | 19.91 | – |
|  | 14.00 | 10.69 | 16.20 | – | 11.64 | 8.40 | 12.73 | – | 8.98 | 5.42 | 8.21 | – | 14.8 | 12.49 | 18.92 | – |
|  | 6.81 | 1.70 | 2.58 | – | 8.56 | 5.32 | 8.06 | – | 10.00 | 6.44 | 9.76 | – | 20.06 | 17.75 | 26.89 | – |
|  | 7.43 | 2.97 | 4.50 | – | 9.59 | 6.35 | 9.62 | – | 9.80 | 6.24 | 9.45 | – | 10.00 | 7.69 | 11.65 | – |
|  | 8.25 | 3.89 | 5.89 | – | 8.77 | 5.53 | 8.38 | – | 10.21 | 6.65 | 10.08 | – | 9.39 | 7.08 | 10.73 | – |
|  | 9.67 | 8.34 | 12.64 | – | 12.26 | 9.02 | 13.67 | – | 11.03 | 7.47 | 11.32 | – | 16.16 | 13.85 | 20.98 | – |
|  | 9.87 | 9.21 | 13.95 | – | 10.82 | 7.58 | 11.48 | – | 9.18 | 5.62 | 8.52 | – | 20.88 | 18.57 | 28.14 | – |
|  | 7.02 | 4.40 | 6.67 | – | 10.62 | 7.38 | 11.18 | – | 12.88 | 9.32 | 14.12 | – | 10.41 | 8.10 | 12.27 | – |
|  | 11.72 | 31.33 | 47.47 | – | 11.23 | 7.99 | 12.11 | – | 17.60 | 14.04 | 21.27 | – | 47.33 | 45.02 | 68.21 | – |
|  | 6.59 | 7.20 | 10.91 | – | 8.77 | 5.53 | 8.38 | – | 27.04 | 23.48 | 35.58 | – | 7.95 | 5.64 | 8.55 | – |
|  | 13.67 | 9.43 | 14.29 | – | 10.21 | 6.97 | 10.56 | – | 8.56 | 5.00 | 7.58 | – | 71.75 | 69.44 | 105.21 | – |
|  | 7.21 | 4.27 | 6.47 | – | 29.10 | 25.86 | 39.18 | – | 14.52 | 10.96 | 16.61 | – | 73.17 | 70.86 | 107.36 | – |
|  | 6.79 | 3.22 | 4.88 | – | 9.80 | 6.56 | 9.94 | – | 40.59 | 37.03 | 56.11 | – | 33.44 | 31.13 | 47.17 | – |
|  | 14.00 | 11.00 | 16.67 | – | 11.12 | 7.88 | 11.94 | – | 11.64 | 8.08 | 12.24 | – | 80.36 | 78.05 | 118.26 | – |
|  | 14.9 | 12.77 | 19.35 | – | 10.41 | 7.17 | 10.86 | – | 9.8 | 6.24 | 9.45 | – | 61.94 | 59.63 | 90.35 | – |
|  | 7.21 | 6.11 | 9.26 | – | 11.44 | 8.2 | 12.42 | – | 18.83 | 15.27 | 23.14 | – | 7.21 | 4.90 | 7.42 | – |
|  | 8.64 | 6.29 | 9.53 | – | 8.56 | 5.32 | 8.06 | – | **248.8** | **208.72** | **316.24** | **+** | 8.64 | 6.33 | 9.59 | – |
|  | 6.59 | 4.30 | 6.52 | – | 9.80 | 6.56 | 9.94 | – | 16.16 | 12.60 | 19.09 | – | 6.59 | 4.28 | 6.48 | – |
|  | 8.44 | 6.22 | 9.42 | – | 11.03 | 7.79 | 11.80 | – | 20.06 | 16.50 | 25.00 | – | 8.44 | 6.13 | 9.29 | – |
|  | 93.33 | 64.74 | 98.09 | – | 28.68 | 25.44 | 38.55 | – | 9.80 | 6.24 | 9.45 | – | 93.33 | 91.02 | 137.91 | – |
|  | 107.73 | 9.87 | 14.95 | – | **387.18** | **230.22** | **348.82** | **+** | 9.39 | 5.83 | 8.83 | – | 107.73 | 105.42 | 159.73 | – |
|  | 81.03 | 70.75 | 107.20 | – | 10.82 | 7.58 | 11.48 | – | 8.36 | 4.80 | 7.27 | – | 81.03 | 78.72 | 119.27 | – |
|  | 76.93 | 75.97 | 115.11 | – | 9.80 | 6.56 | 9.94 | – | 13.08 | 9.52 | 14.42 | – | 76.93 | 74.62 | 113.06 | – |
|  | 14.05 | 13.22 | 20.03 | – | 11.64 | 8.4 | 12.73 | – | 8.36 | 4.80 | 7.27 | – | 14.05 | 11.74 | 17.79 | – |
|  | 68.73 | 59.31 | 89.86 | – | 18.62 | 15.38 | 23.30 | – | 15.34 | 11.78 | 17.85 | – | 68.73 | 66.42 | 100.64 | – |
|  | 72.83 | 43.31 | 65.62 | – | 34.43 | 31.19 | 47.26 | – | 8.56 | 5.00 | 7.58 | – | 72.83 | 70.52 | 106.85 | – |
|  | 79.73 | 53.96 | 81.76 | – | 16.57 | 13.33 | 20.20 | – | 18.56 | 15.00 | 22.73 | – | 79.73 | 77.42 | 117.30 | – |
|  | 74.93 | 58.77 | 89.05 | – | 8.77 | 5.53 | 8.38 | – | 12.88 | 9.32 | 14.12 | – | 74.93 | 72.62 | 110.03 | – |
|  | 83.83 | 49.98 | 75.73 | – | 10.00 | 6.76 | 10.24 | – | 14.52 | 10.96 | 16.61 | – | 83.83 | 81.52 | 123.52 | – |
|  | 18.52 | 15.16 | 22.97 | – | 13.29 | 10.05 | 15.23 | – | 12.26 | 8.70 | 13.18 | – | 18.52 | 16.21 | 24.56 | – |
|  | 15.85 | 13.08 | 19.82 | – | 9.18 | 5.94 | 9.00 | – | 21.5 | 17.94 | 27.18 | – | 15.85 | 13.54 | 20.52 | – |
|  | 55.12 | 42.98 | 65.12 | – | 10.18 | 6.94 | 10.52 | – | 13.7 | 10.14 | 15.36 | – | 55.12 | 52.81 | 80.02 | – |
|  | 27.24 | 23.77 | 36.02 | – | 8.98 | 5.74 | 8.70 | – | 19.45 | 15.89 | 24.08 | – | 27.24 | 24.93 | 37.77 | – |
|  | 68.34 | 60.14 | 91.12 | – | 14.31 | 11.07 | 16.77 | – | 10.21 | 6.65 | 10.08 | – | 68.34 | 66.03 | 100.05 | – |
|  | 8.55 | 8.36 | 12.67 | – | 20.06 | 16.82 | 25.48 | – | 15.13 | 11.57 | 17.53 | – | 13.29 | 10.98 | 16.64 | – |
|  | 19.66 | 14.20 | 21.52 | – | 16.57 | 13.33 | 20.20 | – | 10.00 | 6.44 | 9.76 | – | 21.91 | 19.60 | 29.70 | – |
|  | 75.46 | 52.07 | 78.89 | – | 32.59 | 29.35 | 44.47 | – | 10.62 | 7.06 | 10.70 | – | 11.44 | 9.13 | 13.83 | – |
|  | 17.56 | 22.77 | 34.50 | – | 9.59 | 6.35 | 9.62 | – | 8.77 | 5.21 | 7.89 | – | 13.70 | 11.39 | 17.26 | – |
|  | 20.47 | 16.91 | 25.62 | – | 8.98 | 5.74 | 8.70 | – | 25.81 | 22.25 | 33.71 | – | 10.62 | 8.31 | 12.59 | – |
|  | 9.18 | 5.94 | 9.00 | – | 17.98 | 14.74 | 22.33 | – | 8.77 | 5.21 | 7.89 | – | 8.56 | 6.25 | 9.47 | – |
|  | 12.47 | 9.23 | 13.98 | – | 10.62 | 7.38 | 11.18 | – | 8.56 | 5.00 | 7.58 | – | 9.18 | 6.87 | 10.41 | – |
|  | 8.98 | 5.74 | 8.70 | – | 8.56 | 5.32 | 8.06 | – | 9.18 | 5.62 | 8.52 | – | 18.21 | 15.90 | 24.09 | – |
|  | 11.44 | 8.20 | 12.42 | – | 14.11 | 10.87 | 16.47 | – | 15.75 | 12.19 | 18.47 | – | 13.90 | 11.59 | 17.56 | – |
|  | 12.67 | 9.43 | 14.29 | – | 17.39 | 14.15 | 21.44 | – | 16.16 | 12.60 | 19.09 | – | **248.33** | **198.33** | **300.50** | **+** |
|  | **398.23** | **317.00** | **480.30** | **+** | 38.95 | 35.71 | 54.11 | – | 20.62 | 17.06 | 25.85 | – | 10.21 | 7.9 | 11.97 | – |
|  | 10.62 | 7.38 | 11.18 | – | 34.84 | 31.6 | 47.88 | – | 14.11 | 10.55 | 15.98 | – | 9.39 | 7.08 | 10.73 | – |
|  | 15.13 | 11.89 | 18.02 | – | 8.98 | 5.74 | 8.70 | – | 18.62 | 15.06 | 22.82 | – | 10.62 | 8.31 | 12.59 | – |
|  | 19.04 | 15.8 | 23.94 | – | 9.39 | 6.15 | 9.32 | – | 17.42 | 13.86 | 21.00 | – | 9.18 | 6.87 | 10.41 | – |
|  | 11.23 | 7.99 | 12.11 | – | 22.32 | 19.08 | 28.91 | – | 8.56 | 5.00 | 7.58 | – | 19.8 | 17.49 | 26.50 | – |
|  | 8.77 | 5.53 | 8.38 | – | 13.08 | 9.84 | 14.91 | – | 10.62 | 7.06 | 10.70 | – | 37.51 | 35.2 | 53.33 | – |
|  | 32.38 | 28.82 | 43.67 | – | 10.00 | 6.76 | 10.24 | – | 19.24 | 15.68 | 23.76 | – | 12.88 | 10.57 | 16.02 | – |
|  | 37.72 | 34.16 | 51.76 | – | 12.05 | 8.81 | 13.35 | – | 21.85 | 18.29 | 27.71 | – | 11.23 | 8.92 | 13.52 | – |
|  | 8.36 | 4.80 | 7.27 | – | 10.21 | 6.97 | 10.56 | – | 13.85 | 10.29 | 15.59 | – | 9.39 | 7.08 | 10.73 | – |
|  | 10.21 | 6.65 | 10.08 | – | 9.39 | 6.15 | 9.32 | – | 15.96 | 12.40 | 18.79 | – | 18.56 | 16.25 | 24.62 | – |
|  | 46.75 | 43.19 | 65.44 | – | 14.11 | 10.87 | 16.47 | – | 14.52 | 10.96 | 16.61 | – | 9.56 | 7.25 | 10.98 | – |
|  | 20.27 | 16.71 | 25.32 | – | 8.77 | 5.53 | 8.38 | – | 9.59 | 6.03 | 9.14 | – | 9.39 | 7.08 | 10.73 | – |
|  | 18.21 | 14.65 | 22.20 | – | 20.06 | 16.82 | 25.48 | – | 9.39 | 5.83 | 8.83 | – | 13.39 | 11.08 | 16.79 | – |
|  | 9.39 | 5.83 | 8.83 | – | 8.98 | 5.74 | 8.70 | – | 51.06 | 47.50 | 71.97 | – | 12.88 | 10.57 | 16.02 | – |
|  | 8.77 | 5.21 | 7.89 | – | 10.21 | 6.65 | 10.08 | – | 16.85 | 13.29 | 20.14 | – | 39.56 | 37.25 | 56.44 | – |

Note: C_a_, the concentration of triazophos determined by GC-MS (μg/kg); C_b_, the concentration of triazophos determined by CLEIA (μg/kg); C_c_, the concentration of triazophos corrected correction factor (μg/kg); “+”, positive sample decided by GC-MS; “—”: negative sample decided by GC-MS.
