# Supplementary material for: The Rapid Screening of Triazophos Residues in Agricultural Products by Chemiluminescent Enzyme Immunoassay
Source: PLoS One. 2015 Jul 28;10(7):e0133839. doi: 10.1371/journal.pone.0133839 (PMC4517747; doi:10.1371/journal.pone.0133839)
Supplement: S3 Text — (DOCX) [file pone.0133839.s003.docx]

S3 The determinations of cabbage real samples by GC-MS and CLEIA

| Sample | Ca | Cb | Cc | Results | Ca | Cb | Cc | Results | Ca | Cb | Cc | Result | Ca | Cb | Cc | Results |
| --- | --- | --- | --- | --- | --- | --- | --- | --- | --- | --- | --- | --- | --- | --- | --- | --- |
| cabbage | 6.58 | 4.62 | 5.44 | - | 11.06 | 9.38 | 11.03 | - | 15.47 | 13.28 | 15.62 | - | 16.03 | 14.45 | 17.00 | - |
|  | 9.66 | 7.70 | 9.06 | - | 14.68 | 13.00 | 15.29 | - | 12.80 | 11.61 | 13.66 | - | 13.98 | 12.4 | 14.59 | - |
|  | 9.86 | 7.90 | 9.29 | - | 14.91 | 13.23 | 15.57 | - | 16.70 | 15.51 | 18.25 | - | 15.83 | 14.71 | 17.32 | - |
|  | 14.79 | 12.83 | 15.09 | - | 20.71 | 19.03 | 22.39 | - | 14.39 | 13.20 | 15.53 | - | 10.72 | 9.09 | 11.30 | - |
|  | 47.2 | 45.24 | 53.22 | - | 58.84 | 57.16 | 67.25 | - | 13.57 | 12.38 | 14.56 | - | 15.12 | 14.09 | 16.48 | - |
|  | 69.2 | 67.24 | 79.11 | - | 84.73 | 83.05 | 97.70 | - | 20.96 | 19.77 | 23.26 | - | 48.42 | 47.09 | 55.66 | - |
|  | 7.81 | 5.85 | 6.88 | - | 12.50 | 10.82 | 12.73 | - | 13.77 | 12.58 | 14.80 | - | 34.32 | 33.09 | 39.07 | - |
|  | 23.15 | 21.19 | 24.93 | - | 30.55 | 28.87 | 33.96 | - | 14.03 | 11.84 | 13.93 | - | 14.60 | 13.02 | 15.32 | - |
|  | 6.58 | 4.62 | 5.44 | - | 11.06 | 9.38 | 11.03 | - | 15.47 | 13.28 | 15.62 | - | 16.03 | 14.45 | 17.00 | - |
|  | 43.43 | 38.42 | 50.55 | - | 84.46 | 67.02 | 88.18 | - | 9.82 | 8.07 | 10.62 | - | 11.44 | 9.81 | 11.54 | - |
|  | 98.89 | 56.32 | 74.11 | - | 48.74 | 40.32 | 53.05 | - | 8.77 | 6.58 | 7.74 | - | 8.56 | 6.93 | 8.15 | - |
|  | 48.93 | 39.00 | 51.32 | - | 77.9 | 67.54 | 88.87 | - | 78.32 | 69.43 | 91.36 | - | 25.56 | 23.93 | 28.15 | - |
|  | 17.26 | 15.85 | 18.65 | - | 8.33 | 6.43 | 8.46 | - | 90.30 | 87.29 | 114.86 | - | 17.6 | 15.97 | 18.79 | - |
|  | **567.74** | **442.36** | **520.42** | **+** | 5.98 | 5.3 | 6.97 | - | 89.32 | 57.55 | 75.72 | - | 9.18 | 7.55 | 8.88 | - |
|  | 47.87 | 30.23 | 39.78 | - | 54.22 | 52.03 | 61.21 | - | 33.67 | 34.01 | 44.75 | - | 14.11 | 12.48 | 14.68 | - |
|  | 14.21 | 12.02 | 14.14 | - | 28.33 | 18.00 | 23.68 | - | 22.11 | 21.4 | 28.16 | - | 15.34 | 13.71 | 16.13 | - |
|  | 11.09 | 10.32 | 13.58 | - | 56.68 | 53.49 | 62.93 | - | 37.80 | 11.84 | 15.58 | - | 8.77 | 7.14 | 8.40 | - |
|  | 9.80 | 8.34 | 10.97 | - | 78.21 | 50.34 | 66.24 | - | 56.32 | 47.70 | 62.76 | - | 68.56 | 66.93 | 78.74 | - |
|  | 5.63 | 5.32 | 7.00 | - | 23.34 | 20.55 | 27.04 | - | 88.32 | 83.59 | 109.99 | - | 12.26 | 10.63 | 12.51 | - |
|  | 15.13 | 13.17 | 15.49 | - | 11.44 | 10.13 | 11.92 | - | 11.44 | 9.25 | 10.88 | - | 16.16 | 14.53 | 17.09 | - |
|  | 8.56 | 6.60 | 7.76 | - | 8.56 | 7.25 | 8.53 | - | 8.56 | 6.37 | 7.49 | - | 18.21 | 16.58 | 19.51 | - |
|  | 17.77 | 15.81 | 18.60 | - | 18.56 | 17.25 | 20.29 | - | 48.56 | 46.37 | 54.55 | - | 10.00 | 8.37 | 9.85 | - |
|  | 8.77 | 6.81 | 8.01 | - | 17.6 | 16.29 | 19.16 | - | 17.60 | 15.41 | 18.13 | - | 11.85 | 10.22 | 12.02 | - |
|  | 11.44 | 9.48 | 11.15 | - | 9.18 | 7.87 | 9.26 | - | 9.18 | 6.99 | 8.22 | - | 15.34 | 13.71 | 16.13 | - |
|  | 12.05 | 10.09 | 11.87 | - | 14.11 | 12.80 | 15.06 | - | 14.11 | 11.92 | 14.02 | - | 45.34 | 43.85 | 51.59 | - |
|  | 11.23 | 9.27 | 10.91 | - | 15.34 | 14.03 | 16.51 | - | 15.34 | 13.15 | 15.47 | - | 8.77 | 7.28 | 8.56 | - |
|  | 14.31 | 12.35 | 14.53 | - | 8.77 | 7.46 | 8.78 | - | **109.98** | **88.23** | **103.80** | **+** | 11.85 | 10.36 | 12.19 | - |
|  | 16.37 | 14.41 | 16.95 | - | 8.56 | 7.25 | 8.53 | - | 8.56 | 6.37 | 7.49 | - | 12.05 | 10.56 | 12.42 | - |
|  | 9.80 | 7.84 | 9.22 | - | 12.26 | 10.95 | 12.88 | - | 12.26 | 10.07 | 11.85 | - | 16.98 | 15.49 | 18.22 | - |
|  | 16.98 | 15.02 | 17.67 | - | 16.16 | 14.85 | 17.47 | - | 16.16 | 13.97 | 16.44 | - | 9.39 | 7.9 | 9.29 | - |
|  | 15.34 | 13.38 | 15.74 | - | 18.21 | 16.90 | 19.88 | - | 18.21 | 16.02 | 18.85 | - | 59.39 | 57.9 | 68.12 | - |
|  | 13.23 | 11.27 | 13.26 | - | 10.00 | 8.69 | 10.22 | - | 10.00 | 7.81 | 9.19 | - | 36.68 | 35.19 | 41.40 | - |
|  | 14.23 | 12.27 | 14.44 | - | 11.85 | 10.54 | 12.40 | - | 11.85 | 9.66 | 11.36 | - | 33.6 | 32.11 | 37.78 | - |
|  | 19.85 | 17.89 | 21.05 | - | 15.34 | 13.03 | 15.33 | - | 15.34 | 13.15 | 15.47 | - | 33.39 | 31.90 | 37.53 | - |
|  | 10.06 | 8.10 | 9.53 | - | 17.34 | 15.03 | 17.68 | - | 25.34 | 23.15 | 27.24 | - | 34.83 | 32.34 | 38.05 | - |
|  | 9.59 | 7.63 | 8.98 | - | 8.77 | 6.46 | 7.60 | - | 8.77 | 6.58 | 7.74 | - | 40.17 | 37.68 | 44.33 | - |
|  | 18.36 | 16.40 | 19.29 | - | 11.85 | 9.54 | 11.22 | - | 11.85 | 9.66 | 11.36 | - | 34.01 | 31.52 | 37.08 | - |
|  | 8.36 | 6.40 | 7.53 | - | 52.05 | 49.74 | 58.52 | - | 12.05 | 9.86 | 11.60 | - | 34.42 | 31.93 | 37.56 | - |
|  | 19.36 | 17.40 | 20.47 | - | 16.98 | 16.3 | 19.18 | - | 16.98 | 14.79 | 17.40 | - | 35.86 | 33.37 | 39.26 | - |
|  | 9.18 | 7.22 | 8.49 | - | 9.39 | 8.71 | 10.25 | - | 9.39 | 7.20 | 8.47 | - | 34.22 | 31.73 | 37.33 | - |
|  | 64.56 | 62.60 | 73.65 | - | 39.39 | 38.71 | 45.54 | - | 11.39 | 9.20 | 10.82 | - | 34.01 | 31.52 | 37.08 | - |
|  | 8.77 | 6.81 | 8.01 | - | 9.25 | 7.57 | 8.91 | - | 60.38 | 58.19 | 68.46 | - | 14.76 | 11.87 | 13.96 | - |
|  | 28.56 | 26.60 | 31.29 | - | 6.37 | 4.69 | 5.52 | - | 54.83 | 52.64 | 61.93 | - | 7.57 | 4.68 | 5.51 | - |
|  | 9.39 | 7.43 | 8.74 | - | 46.37 | 44.69 | 52.58 | - | **573.9** | **483.90** | **569.29** | **+** | 8.19 | 5.30 | 6.24 | - |
|  | 19.39 | 17.43 | 20.51 | - | 15.41 | 13.73 | 16.15 | - | 35.24 | 33.05 | 38.88 | - | 9.01 | 6.12 | 7.20 | - |
|  | 9.39 | 7.43 | 8.74 | - | 6.99 | 5.31 | 6.25 | - | 54.01 | 51.82 | 60.96 | - | 10.86 | 7.97 | 9.38 | - |
|  | 14.80 | 12.84 | 15.11 | - | 11.92 | 10.24 | 12.05 | - | 19.55 | 16.36 | 19.25 | - | 11.06 | 8.17 | 9.61 | - |
|  | 54.33 | 52.37 | 61.61 | - | 66.23 | 64.55 | 75.94 | - | 55.24 | 52.05 | 61.24 | - | 8.21 | 6.20 | 7.29 | - |
|  | 67.63 | 65.67 | 77.26 | - | 81.88 | 80.20 | 94.35 | - | **328.9** | **278.66** | **327.84** | **+** | 12.91 | 10.9 | 12.82 | - |
|  | 13.53 | 11.57 | 13.61 | - | 18.23 | 16.55 | 19.47 | - | 24.01 | 20.82 | 24.49 | - | 7.78 | 5.77 | 6.79 | - |
|  | 40.65 | 38.69 | 45.52 | - | 50.14 | 48.46 | 57.01 | - | 37.91 | 34.72 | 40.85 | - | 14.46 | 12.45 | 14.65 | - |
|  | 45.33 | 43.37 | 51.02 | - | 55.64 | 53.96 | 63.49 | - | 13.60 | 10.41 | 12.25 | - | 8.00 | 5.99 | 7.05 | - |
|  | 39.43 | 37.47 | 44.08 | - | 48.70 | 47.02 | 55.32 | - | 12.78 | 9.59 | 11.28 | - | 7.58 | 6.57 | 7.73 | - |
|  | 26.33 | 24.37 | 28.67 | - | 33.29 | 31.61 | 37.19 | - | 40.17 | 36.98 | 43.51 | - | 14.79 | 13.78 | 16.21 | - |
|  | 11.53 | 9.57 | 11.26 | - | 15.88 | 14.20 | 16.70 | - | 16.47 | 13.28 | 15.62 | - | 15.69 | 14.68 | 17.27 | - |
|  | 16.43 | 14.47 | 17.02 | - | 21.64 | 19.96 | 23.49 | - | 13.39 | 11.20 | 13.18 | - | 8.00 | 6.93 | 8.22 | - |
|  | 45.12 | 43.16 | 50.78 | - | 56.40 | 54.72 | 64.37 | - | 23.18 | 20.99 | 24.69 | - | 9.43 | 8.42 | 9.91 | - |
|  | 42.45 | 40.49 | 47.64 | - | 53.26 | 51.58 | 60.68 | - | 14.62 | 12.43 | 14.62 | - | 7.38 | 6.37 | 7.49 | - |
|  | 31.72 | 29.76 | 35.01 | - | 40.63 | 38.95 | 45.83 | - | 19.96 | 17.77 | 20.91 | - | 9.23 | 8.22 | 9.67 | - |
|  | 13.84 | 11.88 | 13.98 | - | 19.60 | 17.92 | 21.08 | - | 13.80 | 11.61 | 13.66 | - | 33.81 | 32.8 | 38.59 | - |
|  | 9.94 | 7.98 | 9.39 | - | 15.01 | 13.33 | 15.68 | - | **111.33** | **100.00** | **117.65** | **+** | 35.24 | 34.23 | 40.27 | - |
|  | 35.15 | 33.19 | 39.05 | - | 44.67 | 42.99 | 50.57 | - | 15.65 | 13.46 | 15.84 | - | 23.19 | 22.18 | 26.09 | - |
|  | 11.26 | 9.30 | 10.94 | - | 16.56 | 14.88 | 17.51 | - | 14.01 | 11.82 | 13.91 | - | 35.04 | 34.03 | 40.04 | - |
|  | 32.06 | 30.10 | 35.41 | - | 41.03 | 35.35 | 41.59 | - | 14.80 | 14.99 | 17.64 | - | 19.93 | 18.52 | 21.79 | - |
|  | 54.16 | 52.20 | 61.41 | - | 67.03 | 65.35 | 76.88 | - | 15.01 | 15.20 | 17.88 | - | 15.21 | 13.80 | 16.24 | - |
|  | 33.86 | 31.90 | 37.53 | - | 41.15 | 39.47 | 46.43 | - | 18.09 | 18.28 | 21.51 | - | 17.06 | 15.65 | 18.41 | - |
|  | 13.15 | 11.19 | 13.16 | - | 16.78 | 15.10 | 17.77 | - | 14.18 | 14.37 | 16.91 | - | **120.47** | **87.43** | **102.86** | **+** |
|  | 6.58 | 4.62 | 5.44 | - | 9.06 | 7.38 | 8.68 | - | 15.83 | 16.02 | 18.85 | - | 14.41 | 13.00 | 15.29 | - |
|  | 6.37 | 4.41 | 5.19 | - | 8.81 | 6.13 | 7.21 | - | 14.18 | 14.37 | 16.91 | - | 19.11 | 17.70 | 20.82 | - |
|  | 10.07 | 8.11 | 9.54 | - | 13.16 | 11.48 | 13.51 | - | 21.17 | 21.36 | 25.13 | - | 13.98 | 12.57 | 14.79 | - |
|  | 13.97 | 12.01 | 14.13 | - | 17.75 | 16.07 | 18.91 | - | 15.62 | 12.43 | 14.62 | - | 21.06 | 19.65 | 23.12 | - |
|  | 36.02 | 34.06 | 40.07 | - | 43.69 | 42.01 | 49.42 | - | 15.01 | 12.82 | 15.08 | - | 14.6 | 13.19 | 15.52 | - |
|  | 13.15 | 11.19 | 13.16 | - | 18.78 | 17.10 | 20.12 | - | 14.03 | 13.84 | 16.28 | - | 14.18 | 12.60 | 14.82 | - |
|  | 16.58 | 14.62 | 17.20 | - | 22.82 | 21.14 | 24.87 | - | 12.80 | 10.61 | 12.48 | - | 21.39 | 19.81 | 23.31 | - |
|  | 29.66 | 27.70 | 32.59 | - | 38.21 | 36.53 | 42.97 | - | 18.34 | 16.15 | 19.00 | - | 22.29 | 20.71 | 24.36 | - |

Note: C_a_, the concentration of triazophos determined by GC-MS (μg/kg); C_b_, the concentration of triazophos determined by CLEIA (μg/kg); C_c_, the concentration of triazophos corrected correction factor (μg/kg); “+”, positive sample decided by GC-MS; “—”: negative sample decided by GC-MS.
