# Supplementary material for: The Rapid Screening of Triazophos Residues in Agricultural Products by Chemiluminescent Enzyme Immunoassay
Source: PLoS One. 2015 Jul 28;10(7):e0133839. doi: 10.1371/journal.pone.0133839 (PMC4517747; doi:10.1371/journal.pone.0133839)
Supplement: S4 Text — (DOCX) [file pone.0133839.s004.docx]

S4 The determinations of zucchinis real samples by GC-MS and CLEIA

| Sample | Ca | Cb | Cc | Results | Ca | Cb | Cc | Results | Ca | Cb | Cc | Result | Ca | Cb | Cc | Results |
| --- | --- | --- | --- | --- | --- | --- | --- | --- | --- | --- | --- | --- | --- | --- | --- | --- |
| zucchinis | 39.73 | 38.42 | 50.55 | - | 9.18 | 7.87 | 10.36 | - | 52.27 | 48.75 | 64.14 | - | 32.10 | 27.15 | 35.72 | - |
|  | 28.3 | 26.99 | 35.51 | - | 8.43 | 7.12 | 9.37 | - | 34.21 | 30.69 | 40.38 | - | 37.64 | 32.69 | 43.01 | - |
|  | 38.9 | 37.59 | 49.46 | - | 22.66 | 21.35 | 28.09 | - | 5.64 | 2.12 | 2.79 | - | 32.1 | 27.15 | 35.72 | - |
|  | 13.29 | 11.98 | 15.76 | - | 91.91 | 90.6 | 119.21 | - | 44.34 | 40.82 | 53.71 | - | 36.00 | 31.05 | 40.86 | - |
|  | 27.38 | 26.07 | 34.30 | - | **243.48** | **242.17** | **318.64** | **+** | 18.43 | 14.91 | 19.62 | - | 36.21 | 31.26 | 41.13 | - |
|  | 12.98 | 11.67 | 15.36 | - | 43.33 | 42.02 | 55.29 | - | 20.63 | 17.11 | 22.51 | - | 33.33 | 28.38 | 37.34 | - |
|  | 23.11 | 21.8 | 28.68 | - | 38.43 | 37.12 | 48.84 | - | 18.32 | 14.80 | 19.47 | - | 23.71 | 18.76 | 24.68 | - |
|  | 34.27 | 32.96 | 43.37 | - | 43.67 | 42.36 | 55.74 | - | 29.77 | 26.25 | 34.54 | - | 23.5 | 18.55 | 24.41 | - |
|  | 13.82 | 12.51 | 16.46 | - | 59.90 | 56.38 | 74.18 | - | 89.76 | 86.24 | 113.47 | - | 26.78 | 21.83 | 28.72 | - |
|  | 10.38 | 9.07 | 11.93 | - | **128.09** | **124.57** | **163.91** | **+** | 82.90 | 79.38 | 104.45 | - | 13.91 | 10.96 | 14.42 | - |
|  | 8.77 | 7.46 | 9.82 | - | 22.04 | 18.52 | 24.37 | - | 52.27 | 48.75 | 64.14 | - | 14.12 | 11.17 | 14.70 | - |
|  | 14.31 | 13.00 | 17.11 | - | 27.58 | 24.06 | 31.66 | - | 57.81 | 54.29 | 71.43 | - | 13.50 | 10.55 | 13.88 | - |
|  | 18.77 | 17.46 | 22.97 | - | 22.04 | 18.52 | 24.37 | - | 52.27 | 48.75 | 64.14 | - | 19.66 | 16.71 | 21.99 | - |
|  | 12.67 | 11.36 | 14.95 | - | 25.94 | 22.67 | 29.83 | - | 56.17 | 52.65 | 69.28 | - | 22.85 | 19.9 | 26.18 | - |
|  | 12.88 | 11.57 | 15.22 | - | 26.15 | 22.88 | 30.11 | - | 56.38 | 52.86 | 69.55 | - | 23.20 | 20.25 | 26.64 | - |
|  | 10.00 | 8.69 | 11.43 | - | 23.27 | 20.00 | 26.32 | - | 53.50 | 49.98 | 65.76 | - | 23.16 | 22.21 | 29.22 | - |
|  | 8.98 | 7.67 | 10.09 | - | 22.25 | 18.98 | 24.97 | - | 43.88 | 40.36 | 53.11 | - | 23.71 | 22.76 | 29.95 | - |
|  | 12.36 | 11.05 | 14.54 | - | 22.04 | 18.77 | 24.70 | - | **109.89** | **106.37** | **139.96** | **+** | 25.15 | 24.2 | 31.84 | - |
|  | 12.05 | 10.74 | 14.13 | - | 25.32 | 22.87 | 30.09 | - | 46.95 | 43.43 | 57.14 | - | 23.30 | 22.35 | 29.41 | - |
|  | 39.02 | 37.71 | 49.62 | - | 22.45 | 20.00 | 26.32 | - | 34.08 | 30.56 | 40.21 | - | 6.53 | 5.58 | 7.34 | - |
|  | 9.39 | 8.08 | 10.63 | - | 12.32 | 9.87 | 12.99 | - | 34.29 | 28.77 | 37.86 | - | 15.50 | 14.55 | 19.14 | - |
|  | 8.77 | 7.46 | 9.82 | - | 22.04 | 19.59 | 25.78 | - | 33.67 | 28.15 | 37.04 | - | 21.04 | 20.09 | 26.43 | - |
|  | 14.93 | 13.62 | 17.92 | - | 28.20 | 25.75 | 33.88 | - | 39.83 | 34.31 | 45.14 | - | 15.50 | 14.55 | 19.14 | - |
|  | 8.12 | 6.81 | 8.96 | - | 21.39 | 18.94 | 24.92 | - | 43.02 | 37.50 | 49.34 | - | 19.40 | 18.45 | 24.28 | - |
|  | 8.47 | 7.16 | 9.42 | - | 21.74 | 19.29 | 25.38 | - | 43.37 | 37.85 | 49.80 | - | 19.61 | 18.66 | 24.55 | - |
|  | 10.37 | 9.06 | 11.92 | - | 21.70 | 19.25 | 25.33 | - | **147.66** | **142.14** | **187.03** | **+** | 16.73 | 15.78 | 20.76 | - |
|  | 8.98 | 7.67 | 10.09 | - | 22.25 | 19.8 | 26.05 | - | 43.88 | 38.36 | 50.47 | - | 15.71 | 14.76 | 19.42 | - |
|  | 18.42 | 17.11 | 22.51 | - | 23.69 | 21.24 | 27.95 | - | 45.32 | 39.80 | 52.37 | - | 15.50 | 14.55 | 19.14 | - |
|  | 16.57 | 15.26 | 20.08 | - | 21.84 | 18.57 | 24.43 | - | 43.47 | 37.95 | 49.93 | - | 18.78 | 17.83 | 23.46 | - |
|  | 9.80 | 8.49 | 11.17 | - | 15.07 | 11.80 | 15.53 | - | 26.70 | 21.18 | 27.87 | - | 15.91 | 14.96 | 19.68 | - |
|  | 18.77 | 17.46 | 22.97 | - | 24.04 | 20.77 | 27.33 | - | 35.67 | 30.15 | 39.67 | - | 16.12 | 15.17 | 19.96 | - |
|  | 24.31 | 23.00 | 30.26 | - | 29.58 | 26.31 | 34.62 | - | 41.21 | 34.69 | 45.64 | - | 15.50 | 14.55 | 19.14 | - |
|  | 18.77 | 17.46 | 22.97 | - | 24.04 | 20.77 | 27.33 | - | 35.67 | 29.15 | 38.36 | - | 20.37 | 19.42 | 25.55 | - |
|  | 22.67 | 21.36 | 28.11 | - | 27.94 | 24.67 | 32.46 | - | 39.57 | 33.05 | 43.49 | - | 11.71 | 10.76 | 14.16 | - |
|  | 22.88 | 21.57 | 28.38 | - | 28.15 | 24.83 | 32.67 | - | 39.78 | 33.26 | 43.76 | - | 12.91 | 11.96 | 15.74 | - |
|  | 20.00 | 18.69 | 24.59 | - | 25.27 | 21.95 | 28.88 | - | 36.90 | 30.38 | 39.97 | - | 25.15 | 24.2 | 31.84 | - |
|  | 18.98 | 17.67 | 23.25 | - | 24.25 | 20.93 | 27.54 | - | 35.88 | 29.36 | 38.63 | - | 23.3 | 22.35 | 29.41 | - |
|  | 18.77 | 17.46 | 22.97 | - | 24.04 | 20.72 | 27.26 | - | 35.67 | 29.15 | 38.36 | - | 16.53 | 15.58 | 20.50 | - |
|  | 22.05 | 20.74 | 27.29 | - | 27.32 | 24 | 31.58 | - | 38.95 | 32.43 | 42.67 | - | 16.77 | 15.82 | 20.82 | - |
|  | 19.18 | 17.87 | 23.51 | - | 24.45 | 21.13 | 27.80 | - | 36.08 | 26.56 | 34.95 | - | 8.21 | 7.26 | 9.55 | - |
|  | 19.39 | 18.08 | 23.79 | - | 24.66 | 21.34 | 28.08 | - | 36.29 | 26.77 | 35.22 | - | 18.36 | 17.41 | 22.91 | - |
|  | **218.77** | **217.46** | **286.13** | **+** | 24.04 | 20.72 | 27.26 | - | 35.67 | 26.15 | 34.41 | - | 9.59 | 8.64 | 11.37 | - |
|  | 24.93 | 23.62 | 31.08 | - | 30.2 | 26.88 | 35.37 | - | **122.34** | **102.82** | **135.29** | **+** | 8.56 | 7.61 | 10.01 | - |
|  | 14.98 | 13.67 | 17.99 | - | 20.25 | 16.98 | 22.34 | - | 31.88 | 22.36 | 29.42 | - | 14.1 | 13.15 | 17.30 | - |
|  | 16.18 | 14.87 | 19.57 | - | 21.45 | 18.18 | 23.92 | - | 33.08 | 23.56 | 31.00 | - | 8.56 | 7.61 | 10.01 | - |
|  | 28.42 | 27.11 | 35.67 | - | 33.69 | 30.42 | 40.03 | - | 45.32 | 35.8 | 47.11 | - | 26.53 | 21.16 | 27.84 | - |
|  | 26.57 | 25.26 | 33.24 | - | 31.84 | 28.57 | 37.59 | - | 43.47 | 33.95 | 44.67 | - | 19.76 | 14.39 | 18.93 | - |
|  | 19.8 | 18.49 | 24.33 | - | 25.07 | 21.8 | 28.68 | - | 36.7 | 33.18 | 43.66 | - | 28.73 | 23.36 | 30.74 | - |
|  | 12.26 | 10.95 | 14.41 | - | 22.26 | 18.99 | 24.99 | - | 26.94 | 23.42 | 30.82 | - | 34.27 | 28.9 | 38.03 | - |
|  | 17.8 | 16.49 | 21.70 | - | 25.54 | 22.27 | 29.30 | - | 28.38 | 24.86 | 32.71 | - | 28.73 | 23.36 | 30.74 | - |
|  | 12.26 | 10.95 | 14.41 | - | 22.67 | 19.4 | 25.53 | - | 12.46 | 8.94 | 11.76 | - | 32.63 | 27.26 | 35.87 | - |
|  | 16.16 | 14.85 | 19.54 | - | 22.88 | 19.61 | 25.80 | - | 12.67 | 9.15 | 12.04 | - | 32.84 | 27.47 | 36.14 | - |
|  | 16.37 | 15.06 | 19.82 | - | **222.26** | **218.99** | **288.14** | **+** | 9.79 | 6.27 | 8.25 | - | 29.96 | 24.59 | 32.36 | - |
|  | 13.49 | 12.18 | 16.03 | - | 28.42 | 25.15 | 33.09 | - | 8.77 | 5.25 | 6.91 | - | 28.94 | 23.57 | 31.01 | - |
|  | 12.47 | 11.16 | 14.68 | - | 18.47 | 15.2 | 20.00 | - | 8.56 | 5.04 | 6.63 | - | 14.72 | 11.35 | 14.93 | - |
|  | 12.26 | 10.95 | 14.41 | - | 19.67 | 16.4 | 21.58 | - | 11.84 | 8.32 | 10.95 | - | 14.77 | 11.4 | 15.00 | - |
|  | 15.54 | 14.23 | 18.72 | - | 31.91 | 28.64 | 37.68 | - | 8.97 | 5.45 | 7.17 | - | 10.37 | 7 | 9.21 | - |
|  | 12.67 | 11.36 | 14.95 | - | 30.06 | 26.79 | 35.25 | - | 9.18 | 5.66 | 7.45 | - | 14.38 | 11.01 | 14.49 | - |
|  | 12.88 | 11.57 | 15.22 | - | 23.29 | 20.02 | 26.34 | - | 9.38 | 5.86 | 7.71 | - | 16.39 | 13.02 | 17.13 | - |
|  | 12.26 | 10.95 | 14.41 | - | 26.73 | 23.46 | 30.87 | - | 28.73 | 25.21 | 33.17 | - | 10.04 | 6.67 | 8.78 | - |
|  | 18.42 | 17.11 | 22.51 | - | 32.27 | 29 | 38.16 | - | 32.01 | 28.49 | 37.49 | - | 16.78 | 13.41 | 17.64 | - |
|  | 11.61 | 10.30 | 13.55 | - | 26.73 | 23.46 | 30.87 | - | 29.14 | 25.62 | 33.71 | - | 10.06 | 6.69 | 8.80 | - |
|  | 11.96 | 10.65 | 14.01 | - | 30.63 | 27.36 | 36.00 | - | 29.35 | 25.83 | 33.99 | - | 7.19 | 3.82 | 5.03 | - |
|  | 11.92 | 10.61 | 13.96 | - | **126.84** | **123.57** | **162.59** | **+** | 28.73 | 25.21 | 33.17 | - | 17.4 | 14.03 | 18.46 | - |
|  | 12.47 | 11.16 | 14.68 | - | 27.96 | 25.69 | 33.80 | - | 34.89 | 31.37 | 41.28 | - | **88.78** | **81.41** | **107.12** | **+** |
|  | 21.91 | 20.60 | 27.11 | - | 26.94 | 24.67 | 32.46 | - | 24.94 | 21.42 | 28.18 | - | 19.03 | 11.66 | 15.34 | - |
|  | 22.67 | 21.36 | 28.11 | - | 27.94 | 24.67 | 32.46 | - | 39.57 | 33.05 | 43.49 | - | 11.71 | 10.76 | 14.16 | - |
|  | 20.06 | 18.75 | 24.67 | - | 26.73 | 24.46 | 32.18 | - | 11.03 | 9.51 | 12.51 | - | 20.47 | 17.1 | 22.50 | - |
|  | 13.29 | 11.98 | 15.76 | - | 30.01 | 27.74 | 36.50 | - | 10.82 | 9.3 | 12.24 | - | 18.62 | 15.25 | 20.07 | - |
|  | 22.26 | 20.95 | 27.57 | - | 27.14 | 24.87 | 32.72 | - | 14.1 | 12.58 | 16.55 | - | 9.85 | 6.48 | 8.53 | - |
|  | 27.8 | 26.49 | 34.86 | - | 27.35 | 25.08 | 33.00 | - | 11.23 | 9.71 | 12.78 | - | 10.82 | 7.45 | 9.80 | - |
|  | 22.26 | 20.95 | 27.57 | - | 26.73 | 24.46 | 32.18 | - | 26.95 | 23.43 | 30.83 | - | 16.36 | 12.99 | 17.09 | - |
|  | 26.16 | 24.85 | 32.70 | - | 32.89 | 31.62 | 41.61 | - | 30.23 | 26.71 | 35.14 | - | 10.82 | 7.45 | 9.80 | - |
|  | 26.37 | 25.06 | 32.97 | - | 26.08 | 24.81 | 32.64 | - | 27.36 | 23.84 | 31.37 | - | 14.72 | 11.35 | 14.93 | - |
|  | 23.49 | 22.18 | 29.18 | - | 26.43 | 25.16 | 33.11 | - | 27.57 | 24.05 | 31.64 | - | 14.93 | 11.56 | 15.21 | - |
|  | 22.47 | 21.16 | 27.84 | - | 26.39 | 25.12 | 33.05 | - | **226.95** | **223.43** | **293.99** | **+** | 12.05 | 8.68 | 11.42 | - |

Note: C_a_, the concentration of triazophos determined by GC-MS (μg/kg); C_b_, the concentration of triazophos determined by CLEIA (μg/kg); C_c_, the concentration of triazophos corrected correction factor (μg/kg); “+”, positive sample decided by GC-MS; “—”: negative sample decided by GC-MS.
