# Supplementary material for: The Rapid Screening of Triazophos Residues in Agricultural Products by Chemiluminescent Enzyme Immunoassay
Source: PLoS One. 2015 Jul 28;10(7):e0133839. doi: 10.1371/journal.pone.0133839 (PMC4517747; doi:10.1371/journal.pone.0133839)
Supplement: S5 Text — (DOCX) [file pone.0133839.s005.docx]

S5 The determinations of rice real samples by GC-MS and CLEIA

| Sample | Ca | Cb | Cc | Results | Ca | Cb | Cc | Results | Ca | Cb | Cc | Result | Ca | Cb | Cc | Results |
| --- | --- | --- | --- | --- | --- | --- | --- | --- | --- | --- | --- | --- | --- | --- | --- | --- |
| rice | 18.81 | 28.60 | 31.43 | - | 21.67 | 18.46 | 20.29 | - | **120.42** | **111.90** | **122.97** | **+** | 11.15 | 5.09 | 5.59 | - |
|  | 19.23 | 29.02 | 31.89 | - | 22.09 | 18.88 | 20.75 | - | 12.17 | 6.11 | 6.71 | - | 12.23 | 12.35 | 13.57 | - |
|  | 14.39 | 24.18 | 26.57 | - | 14.04 | 10.83 | 11.90 | - | 10.94 | 4.88 | 5.36 | - | 14.65 | 13.13 | 14.43 | - |
|  | 14.50 | 24.29 | 26.69 | - | 14.45 | 14.24 | 15.65 | - | 16.48 | 10.42 | 11.45 | - | 12.52 | 11.00 | 12.09 | - |
|  | 15.94 | 25.73 | 28.27 | - | 14.86 | 14.65 | 16.10 | - | 12.17 | 6.11 | 6.71 | - | 13.54 | 12.02 | 13.21 | - |
|  | 16.35 | 26.14 | 28.73 | - | 13.64 | 13.43 | 14.76 | - | 13.61 | 7.55 | 8.30 | - | 12.31 | 10.79 | 11.86 | - |
|  | 18.20 | 27.99 | 30.76 | - | 14.04 | 13.83 | 15.20 | - | 10.94 | 8.88 | 9.76 | - | 17.85 | 16.33 | 17.95 | - |
|  | 11.42 | 21.21 | 23.31 | - | 13.21 | 13.00 | 14.29 | - | 10.53 | 8.47 | 9.31 | - | 13.54 | 12.02 | 13.21 | - |
|  | 11.84 | 21.63 | 23.77 | - | 20.20 | 19.99 | 21.97 | - | 13.76 | 11.70 | 12.86 | - | 14.98 | 13.46 | 14.79 | - |
|  | 9.23 | 6.98 | 7.87 | - | 5.64 | 4.98 | 5.47 | - | 42.05 | 40.53 | 44.54 | - | 13.89 | 11.83 | 13.00 | - |
|  | 8.90 | 7.98 | 8.87 | - | 34.32 | 35.79 | 39.33 | - | **55.31** | **53.79** | **59.11** | **+** | 14.30 | 12.24 | 13.45 | - |
|  | 9.80 | 8.98 | 9.87 | - | 34.78 | 50.32 | 55.30 | - | 38.08 | 36.56 | 40.18 | - | 14.71 | 12.65 | 13.90 | - |
|  | 17.23 | 14.86 | 16.33 | - | **143.43** | **138.42** | **152.11** | **+** | 37.90 | 36.38 | 39.98 | - | 12.89 | 10.83 | 11.90 | - |
|  | 19.32 | 16.95 | 18.63 | - | 28.01 | 15.08 | 16.57 | - | 32.34 | 30.82 | 33.87 | - | 17.89 | 15.83 | 17.39 | - |
|  | 48.97 | 44.60 | 49.01 | - | 18.79 | 16.91 | 18.58 | - | 11.81 | 11.93 | 13.11 | - | 13.06 | 11.00 | 12.08 | - |
|  | 10.34 | 7.97 | 8.76 | - | 8.93 | 7.41 | 8.14 | - | 22.70 | 20.49 | 22.52 | - | 20.05 | 14.99 | 16.47 | - |
|  | 38.74 | 36.37 | 39.97 | - | 16.82 | 15.30 | 16.81 | - | 12.53 | 16.81 | 18.47 | - | 14.50 | 9.44 | 10.37 | - |
|  | 47.98 | 41.61 | 45.73 | - | 7.37 | 6.90 | 7.58 | - | 18.07 | 20.35 | 22.36 | - | 9.37 | 4.31 | 4.73 | - |
|  | 29.47 | 27.10 | 29.78 | - | 36.12 | 37.45 | 41.15 | - | 13.76 | 16.04 | 17.63 | - | 10.39 | 5.33 | 5.85 | - |
|  | 14.26 | 13.98 | 15.36 | - | 17.52 | 21.40 | 23.52 | - | 15.20 | 17.48 | 19.21 | - | 12.16 | 7.10 | 7.80 | - |
|  | 14.67 | 14.39 | 15.81 | - | 17.93 | 21.81 | 23.97 | - | 12.53 | 14.81 | 16.27 | - | 14.70 | 9.64 | 10.59 | - |
|  | 15.08 | 14.80 | 16.26 | - | 18.34 | 22.22 | 24.42 | - | 12.12 | 20.40 | 22.42 | - | 10.39 | 5.33 | 5.85 | - |
|  | 14.98 | 14.70 | 16.15 | - | 12.52 | 16.40 | 18.02 | - | 12.12 | 10.76 | 11.82 | - | 11.83 | 9.77 | 10.73 | - |
|  | 14.26 | 13.98 | 15.36 | - | 17.52 | 21.40 | 23.52 | - | 14.70 | 13.34 | 14.66 | - | 9.16 | 7.10 | 7.80 | - |
|  | 13.43 | 13.15 | 14.45 | - | 16.69 | 20.57 | 22.60 | - | 18.61 | 18.26 | 20.07 | - | 9.83 | 8.31 | 9.13 | - |
|  | 20.42 | 20.14 | 22.13 | - | 23.68 | 27.56 | 30.29 | - | 18.40 | 18.05 | 19.84 | - | 18.79 | 20.67 | 22.71 | - |
|  | 14.87 | 19.15 | 21.04 | - | 18.13 | 20.01 | 21.99 | - | 19.02 | 18.67 | 20.52 | - | 24.33 | 26.21 | 28.80 | - |
|  | 9.74 | 14.02 | 15.41 | - | 16.00 | 17.88 | 19.65 | - | 19.84 | 16.49 | 18.12 | - | 20.02 | 21.90 | 24.07 | - |
|  | 10.76 | 15.04 | 16.53 | - | 17.02 | 18.90 | 20.77 | - | 13.54 | 12.48 | 13.71 | - | 21.46 | 23.34 | 25.65 | - |
|  | 26.32 | 24.80 | 27.25 | - | 16.82 | 15.30 | 16.81 | - | 14.16 | 13.10 | 14.40 | - | **100.32** | **89.44** | **98.29** | **+** |
|  | 30.66 | 29.14 | 32.02 | - | 11.27 | 9.75 | 10.71 | - | 14.98 | 13.92 | 15.30 | - | 18.38 | 16.50 | 18.13 | - |
|  | 13.49 | 12.43 | 13.66 | - | 9.14 | 7.62 | 8.37 | - | 16.83 | 15.77 | 17.33 | - | 8.38 | 6.50 | 7.14 | - |
|  | 10.66 | 9.14 | 10.04 | - | 10.16 | 8.64 | 9.49 | - | 17.03 | 15.97 | 17.55 | - | 17.56 | 15.68 | 17.23 | - |
|  | 11.07 | 9.55 | 10.49 | - | 8.93 | 7.41 | 8.14 | - | 18.47 | 16.41 | 18.03 | - | 21.47 | 19.59 | 21.53 | - |
|  | 11.48 | 9.96 | 10.95 | - | 14.47 | 12.95 | 14.23 | - | 8.52 | 7.00 | 7.69 | - | 21.26 | 19.38 | 21.30 | - |
|  | 10.66 | 9.14 | 10.04 | - | 10.16 | 8.64 | 9.49 | - | 28.52 | 27.00 | 29.67 | - | 21.88 | 20.00 | 21.98 | - |
|  | 30.66 | 29.14 | 32.02 | - | 11.60 | 10.08 | 11.08 | - | 7.70 | 6.18 | 6.79 | - | 13.95 | 11.89 | 13.07 | - |
|  | 8.75 | 6.69 | 7.35 | - | **98.09** | **92.33** | **101.46** | **+** | 11.61 | 10.09 | 11.09 | - | 14.37 | 12.31 | 13.53 | - |
|  | 15.25 | 14.19 | 15.59 | - | 18.88 | 16.82 | 18.48 | - | 11.40 | 9.88 | 10.86 | - | 9.58 | 7.52 | 8.26 | - |
|  | 11.33 | 10.27 | 11.28 | - | 20.73 | 18.67 | 20.52 | - | 12.02 | 10.50 | 11.54 | - | 9.99 | 7.93 | 8.71 | - |
|  | 12.31 | 10.25 | 11.26 | - | 22.70 | 20.82 | 22.88 | - | 12.84 | 11.32 | 12.44 | - | 10.40 | 8.34 | 9.16 | - |
|  | 21.69 | 21.34 | 23.45 | - | 24.55 | 22.67 | 24.91 | - | 17.69 | 16.17 | 17.77 | - | 9.18 | 7.12 | 7.82 | - |
|  | 9.23 | 6.98 | 7.87 | - | 5.64 | 4.98 | 5.47 | - | 42.05 | 40.53 | 44.54 | - | 13.89 | 11.83 | 13.00 | - |
|  | 21.89 | 20.92 | 22.99 | - | 24.75 | 22.87 | 25.13 | - | 14.89 | 13.37 | 14.69 | - | 9.58 | 7.52 | 8.26 | - |
|  | 23.33 | 17.36 | 19.08 | - | 26.19 | 24.31 | 26.71 | - | 16.33 | 14.81 | 16.27 | - | 8.75 | 6.69 | 7.35 | - |
|  | 23.74 | 22.77 | 25.02 | - | 26.60 | 25.71 | 28.25 | - | 16.74 | 15.22 | 16.73 | - | 15.74 | 13.68 | 15.03 | - |
|  | 25.59 | 24.62 | 27.05 | - | 28.45 | 27.56 | 30.29 | - | 18.59 | 17.07 | 18.76 | - | 10.19 | 8.13 | 8.93 | - |
|  | 18.81 | 17.84 | 19.60 | - | 21.67 | 20.78 | 22.84 | - | 11.81 | 10.29 | 11.31 | - | 8.06 | 6.00 | 6.59 | - |
|  | 19.23 | 17.96 | 19.74 | - | 22.09 | 21.20 | 23.30 | - | 12.23 | 10.71 | 11.77 | - | 9.08 | 7.02 | 7.71 | - |
|  | 14.26 | 12.99 | 14.27 | - | 17.52 | 16.63 | 18.27 | - | 10.66 | 9.14 | 10.04 | - | 8.25 | 6.19 | 6.80 | - |
|  | 14.67 | 13.40 | 14.73 | - | 17.93 | 17.04 | 18.73 | - | 11.07 | 9.55 | 10.49 | - | 13.79 | 11.73 | 12.89 | - |
|  | 15.08 | 13.81 | 15.18 | - | 18.34 | 17.45 | 19.18 | - | 11.48 | 9.96 | 10.95 | - | 9.48 | 7.42 | 8.15 | - |
|  | 13.26 | 11.99 | 13.18 | - | 17.52 | 16.63 | 18.27 | - | 10.66 | 9.14 | 10.04 | - | 10.92 | 8.86 | 9.74 | - |
|  | 22.26 | 27.53 | 30.25 | - | 25.52 | 24.63 | 27.07 | - | 18.66 | 17.14 | 18.84 | - | 8.25 | 6.19 | 6.80 | - |
|  | 13.43 | 18.70 | 20.55 | - | 16.69 | 15.80 | 17.36 | - | 9.83 | 8.31 | 9.13 | - | 7.84 | 5.78 | 6.35 | - |
|  | 20.42 | 25.69 | 28.23 | - | 23.68 | 22.79 | 25.04 | - | **87.32** | **81.38** | **89.43** | **+** | 7.84 | 5.78 | 6.35 | - |
|  | 14.87 | 20.14 | 22.13 | - | 18.13 | 16.78 | 18.44 | - | 11.27 | 9.75 | 10.71 | - | 7.02 | 4.96 | 5.45 | - |
|  | 9.74 | 15.01 | 16.49 | - | 16.00 | 14.65 | 16.10 | - | 9.14 | 7.62 | 8.37 | - | 10.93 | 8.87 | 9.75 | - |
|  | 10.76 | 16.03 | 17.62 | - | 17.02 | 15.67 | 17.22 | - | 10.16 | 8.64 | 9.49 | - | 10.72 | 8.66 | 9.52 | - |
|  | 12.53 | 17.80 | 19.56 | - | 18.79 | 17.44 | 19.16 | - | 8.93 | 7.41 | 8.14 | - | 11.34 | 9.28 | 10.20 | - |
|  | 18.07 | 23.34 | 25.65 | - | 24.33 | 22.98 | 25.25 | - | 14.47 | 12.95 | 14.23 | - | 12.16 | 10.10 | 11.10 | - |
|  | 13.76 | 19.03 | 20.91 | - | 20.02 | 18.67 | 20.52 | - | 10.16 | 8.64 | 9.49 | - | 19.39 | 17.33 | 19.04 | - |
|  | 15.20 | 20.47 | 22.49 | - | 21.46 | 21.25 | 23.35 | - | 11.60 | 10.08 | 11.08 | - | 18.38 | 18.17 | 19.97 | - |
|  | 12.53 | 17.80 | 19.56 | - | 18.79 | 18.58 | 20.42 | - | 8.93 | 7.41 | 8.14 | - | 24.88 | 24.67 | 27.11 | - |
|  | 12.12 | 17.39 | 19.11 | - | 19.59 | 17.53 | 19.26 | - | 8.52 | 7.00 | 7.69 | - | 17.56 | 17.35 | 19.07 | - |
|  | 18.62 | 23.89 | 26.25 | - | 21.03 | 18.97 | 20.85 | - | 15.02 | 13.50 | 14.84 | - | 21.47 | 21.26 | 23.36 | - |
|  | 14.70 | 23.49 | 25.81 | - | 21.44 | 19.38 | 21.30 | - | 7.70 | 6.18 | 6.79 | - | 21.26 | 19.05 | 20.93 | - |
|  | 18.61 | 27.40 | 30.11 | - | 23.29 | 21.23 | 23.33 | - | 11.61 | 10.09 | 11.09 | - | 21.88 | 19.67 | 21.62 | - |
|  | 18.40 | 27.19 | 29.88 | - | 16.51 | 14.45 | 15.88 | - | 11.40 | 9.88 | 10.86 | - | **134.98** | **125.46** | **137.87** | **+** |
|  | 19.02 | 27.81 | 30.56 | - | 16.93 | 14.87 | 16.34 | - | 12.02 | 10.50 | 11.54 | - | 24.55 | 22.34 | 24.55 | - |
|  | 19.84 | 28.63 | 31.46 | - | 12.67 | 11.61 | 12.76 | - | 12.84 | 12.96 | 14.24 | - | 24.75 | 22.54 | 24.77 | - |
|  | 21.69 | 30.48 | 33.49 | - | 13.08 | 12.02 | 13.21 | - | 14.69 | 14.81 | 16.27 | - | 26.19 | 22.98 | 25.25 | - |
|  | 21.89 | 30.68 | 33.71 | - | **56.22** | **54.70** | **60.11** | **+** | 14.89 | 15.01 | 16.49 | - | 26.60 | 23.39 | 25.70 | - |
|  | 23.33 | 32.12 | 35.30 | - | 12.27 | 11.21 | 12.32 | - | 16.33 | 16.45 | 18.08 | - | 11.84 | 10.78 | 11.85 | - |
|  | 23.74 | 32.53 | 35.75 | - | 12.67 | 11.61 | 12.76 | - | 16.74 | 16.86 | 18.53 | - | 18.83 | 17.77 | 19.53 | - |
|  | 25.59 | 34.38 | 37.78 | - | 28.45 | 25.24 | 27.74 | - | 18.59 | 18.71 | 20.56 | - | 13.28 | 12.22 | 13.43 | - |
|  | 21.89 | 20.92 | 22.99 | - | 24.75 | 22.87 | 25.13 | - | 14.89 | 13.37 | 14.69 | - | 9.58 | 7.52 | 8.26 | - |

Note: C_a_, the concentration of triazophos determined by GC-MS (μg/kg); C_b_, the concentration of triazophos determined by CLEIA (μg/kg); C_c_, the concentration of triazophos corrected correction factor (μg/kg); “+”, positive sample decided by GC-MS; “—”: negative sample decided by GC-MS.
